# An autocatalytic CRISPR-Cas amplification effect propelled by the LNA-modified split activators for DNA sensing

**DOI:** 10.1093/nar/gkae176

**Published:** 2024-03-13

**Authors:** Ke Sun, Lei Pu, Chuan Chen, Mutian Chen, Kaiju Li, Xinqiong Li, Huanqing Li, Jia Geng

**Affiliations:** Department of Laboratory Medicine, State Key Laboratory of Biotherapy and Clinical Laboratory Medicine Research Center, West China Hospital, Sichuan University, Chengdu, 610041 Chengdu, China; Tianfu Jincheng Laboratory, City of Future Medicine, Chengdu 641400, China; Department of Laboratory Medicine, State Key Laboratory of Biotherapy and Clinical Laboratory Medicine Research Center, West China Hospital, Sichuan University, Chengdu, 610041 Chengdu, China; Department of Laboratory Medicine, State Key Laboratory of Biotherapy and Clinical Laboratory Medicine Research Center, West China Hospital, Sichuan University, Chengdu, 610041 Chengdu, China; School of Pharmacy, North Sichuan Medical College, 637000 Nanchong, China; Department of Laboratory Medicine, State Key Laboratory of Biotherapy and Clinical Laboratory Medicine Research Center, West China Hospital, Sichuan University, Chengdu, 610041 Chengdu, China; Department of Laboratory Medicine, State Key Laboratory of Biotherapy and Clinical Laboratory Medicine Research Center, West China Hospital, Sichuan University, Chengdu, 610041 Chengdu, China; Department of Laboratory Medicine, State Key Laboratory of Biotherapy and Clinical Laboratory Medicine Research Center, West China Hospital, Sichuan University, Chengdu, 610041 Chengdu, China; Department of Laboratory Medicine, State Key Laboratory of Biotherapy and Clinical Laboratory Medicine Research Center, West China Hospital, Sichuan University, Chengdu, 610041 Chengdu, China; Department of Laboratory Medicine, State Key Laboratory of Biotherapy and Clinical Laboratory Medicine Research Center, West China Hospital, Sichuan University, Chengdu, 610041 Chengdu, China; Tianfu Jincheng Laboratory, City of Future Medicine, Chengdu 641400, China

## Abstract

CRISPR-Cas systems with dual functions offer precise sequence-based recognition and efficient catalytic cleavage of nucleic acids, making them highly promising in biosensing and diagnostic technologies. However, current methods encounter challenges of complexity, low turnover efficiency, and the necessity for sophisticated probe design. To better integrate the dual functions of Cas proteins, we proposed a novel approach called CRISPR-Cas Autocatalysis Amplification driven by LNA-modified Split Activators (CALSA) for the highly efficient detection of single-stranded DNA (ssDNA) and genomic DNA. By introducing split ssDNA activators and the site-directed *trans*-cleavage mediated by LNA modifications, an autocatalysis-driven positive feedback loop of nucleic acids based on the LbCas12a system was constructed. Consequently, CALSA enabled one-pot and real-time detection of genomic DNA and cell-free DNA (cfDNA) from different tumor cell lines. Notably, CALSA achieved high sensitivity, single-base specificity, and remarkably short reaction times. Due to the high programmability of nucleic acid circuits, these results highlighted the immense potential of CALSA as a powerful tool for cascade signal amplification. Moreover, the sensitivity and specificity further emphasized the value of CALSA in biosensing and diagnostics, opening avenues for future clinical applications.

## Graphical Abstract

**Graphical Abstract:**
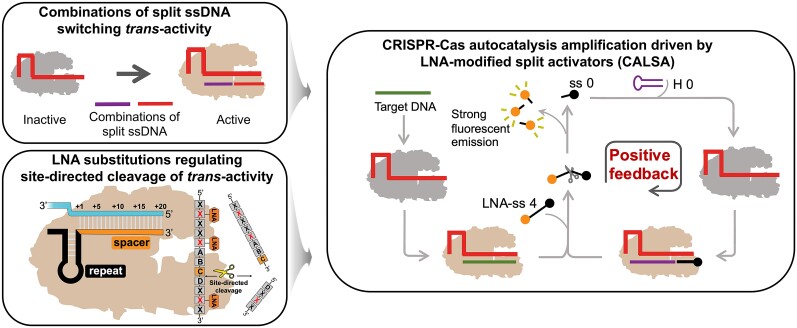

## Introduction

Signal amplification based on molecular biology and biochemical reactions has garnered significant interest and demonstrated considerable potential in the fields of biosensing and bioengineering due to the programmable and autonomous interactions of DNA/RNA and their diverse enzymatic transformations (1–5). As compelling alternatives to the gold-standard thermocycling-required polymerase chain reaction (PCR) (6), some isothermal signal amplification approaches (7) including polymerase and nickase-involved DNA circuit (8,9), hybridization chain reaction (10), catalytic hairpin assembly (2,11) and entropy-driven catalysis (12) have promoted numerous applications of biosensing in clinical and laboratory settings towards rapidity, cost-effectiveness, high efficiency, integration, and versatility. However, despite their numerous benefits, these isothermal methods possess inherent limitations. Most of them rely on ssDNA rather than untreated genomic double-stranded DNA (dsDNA) as the input signal and circuit elements for amplification (13). This restriction hampers the detection of a wide range of clinical targets. Furthermore, many of these methods involve multistep-reaction circuits and exhibit low turnovers, thereby impeding convenience and amplification efficiency (14). Consequently, there is a pressing need to develop new signal amplification schemes that employ novel circuit modules and mechanisms, enabling sensitive responses to various signal inputs and expanding the practical utility of diagnostics.

The Clustered Regularly Interspaced Short Palindromic Repeats (CRISPR) system is a prokaryotic adaptive immune system that plays a defensive role in prokaryotic immunity by targeting genomic loci and degrading invading nucleic acids (15). The CRISPR-associated (Cas) proteins, which enable precise sequence-based recognition and efficient catalytic cleavage of nucleic acids, have significantly expanded our toolkits for highly specific gene editing (16,17). Recently, it has been discovered that some Cas12 or Cas13 family member proteins possess target loading-activated noncanonical *trans*-cleavage endonuclease activity towards collateral ssDNA and dsDNA or RNA, known as *trans* cleavage (18–21). Leveraging the high turnovers of *trans* cleavage (*k*_cat_/*K*_M_ > 10^6^ s^−1^ M^−1^) on ssDNA probes (22,23) and low background leaks of dsDNA probes (20), numerous bio-catalytic signal amplification methods have been established to develop next-generation diagnostic technologies for ultrasensitive nucleic acid detection. For instance, by integrating with extra exponential amplification based on polymerase and linear signal amplification of *trans*-cleavage of Cas proteins, innovative methods like SHERLOCK (22,24), DETECTR (25), HOLMES (26) and others (14,23) have been developed for the detection of target genes and extended non-nucleic acid analytes with attomolar sensitivity and single-base mismatch specificity. While the introduction of pre-nucleic acid amplification strategies such as loop-mediated isothermal amplification (LAMP) (27,28) and recombinase polymerase amplification (RPA) (29,30) can further enhance the utility of Cas-based methodologies and significantly improve sensitivity, these assays still have notable disadvantages. The complex components in the reaction make the detection system intricate, and false results may also be induced. As aforementioned Cas proteins, such as Cas12a, exhibit dual functions of precise sequence-based recognition and efficient catalytic cleavage of nucleic acids (18,31). This characteristic theoretically makes the Cas12a a potentially programmable platform to integrate into autocatalysis-driven nucleic acid circuits, providing a simpler amplification strategy with comparable ultrasensitivity (32).

In recent developments, a *de novo* method called CONAN utilizes Cas12a autocatalysis-driven artificial reaction networks to create a positive feedback circuit with exponential dynamics, and thereby showcase great promise in biosensing applications (13). This method represents a highly successful implementation of the CRISPR/Cas system for self-amplified positive feedback signal amplification. However, despite the unprecedented advancements achieved through this approach, it also faces challenges related to the exponential dynamics of signals, which are hindered by the heavy reliance on changes in the secondary structure of nucleic acid elements to initiate the reaction. Additionally, the design of sophisticated RNA/DNA hybrid probes further adds complexity and raises the threshold for expanding the utility of this method.

Thus, this study aimed to circumvent the aforementioned limitations by introducing split ssDNA activators and the site-directed *trans*-cleavage mediated by LNA modifications into the LbCas12a system, and develop an idealized CRISPR-Cas autocatalysis amplification method which was driven by the LNA-modified split activators for DNA diagnostics (CALSA). CALSA utilized LNA-modified split activators and natural split ssDNA with hairpin structures as key elements for signal amplification, effectively repurposing the LbCas12a system into programmable and autocatalysis-driven nucleic acid circuits. In CALSA, trace amounts of activation triggered an explosive amplification through a positive feedback loop of signal elements with self-reporting capability. This approach achieved a consummate integration of the dual functions of stringent target recognition and efficient *trans*-cleavage activity offered by the LbCas12a system. CALSA enabled one-enzyme, one-pot, and real-time detection of genomic DNA and cell-free DNA (cfDNA) with high sensitivity, single-base specificity, and within a short timeframe of 1 hour. To validate its versatility and analytical performance, synthetic genes (dsDNA) and cfDNA (ssDNA) from different tumor cell lines (MCF-7, HeLa and HEK293T) were employed. In summary, CALSA represented a powerful and promising tool in the field of highly sensitive and specific nucleic acid diagnostics. It successfully addressed the limitations associated with signal amplification methods by leveraging the unique features of the LbCas12a system and incorporating split activators and LNA-mediated site-directed cleavage.

## Materials and methods

### Materials

LbCas12a (Cpf1), AsCas12a, FnCas12a and RNA inhibitor were purchased from New England Biolabs Co. Ltd (NEB, USA). *N*,*N*,*N*',*N*'-Tetramethylethylenediamine (TEMED), urea, ammonium persulphate (APS), 40% acrylamide/bis-acrylamide (19:1), Tris borate EDTA (TBE), 2 × TBE–urea sample buffer and Ezup cfDNA Extraction Kit were obtained from Sangon Biotech Co. Ltd (Shanghai, China). Dulbecco's modified Eagle's medium (DMEM), Minimum Essential Medium (MEM) and fetal bovine serum (FBS) were purchased from Invitrogen (Gibco, USA). Trypsin and dimethyl sulfoxide (DMSO) were purchased from Sigma-Aldrich (St Louis, MO, USA). Human breast cancer cell line (MCF-7), human cervical cancer cell line (HeLa) and human embryonic kidney cancer cell line (HEK293T) were obtained from the Cell Bank of Type Culture Collection of the Chinese Academy of Sciences (Shanghai, China). To create and maintain a ribonuclease-free environment, all the solutions containing RNAs were prepared with RNase-free water.

### Oligonucleotides

All DNA and RNA strands (HPLC purified) used in this work were ordered from Sangon Biotech Co. Ltd (Shanghai, China). DNA strands were dissolved in RNase-free water at a final concentration of 100 μM. All the hairpin DNA were prepared by hybridization of a single strand DNA (ssDNA) at 95°C for 5 min in PBS buffer, followed by gradient cooling to 25°C in PCR equipment (SimpliAmp, ThermoFisher, America). The annealed hairpin DNA were stored at −20°C. All the crRNA used in this work were dissolved in RNase-free water at a final concentration of 20 μM and split into aliquots storage at −20°C. The sequences of all oligonucleotides were listed in Supplementary Tables S1-S14.

### Fluorescence measurement in Cas12a reaction

The fluorescence intensity tests were carried out on a 386-well black clear-bottom plate (Corning) with the Cytation™ 3 machine (BioTek Instruments, USA). FAM was excited at 492 nm, and its emission at 518 nm was recorded.

For assays with single ssDNA or dsDNA as an activator, the Cas12a/crRNA complex was pre-incubated for 5 min at 37°C through mixing a final concentration of 50 nM LbCas12a, 50 nM crRNA and various concentrations of activators. The reaction was initiated by adding the final concentration of 200 nM Probe DNA. Reactions were performed in three replicates (20 μl) at 37°C for 3 h and fluorescence measurements were acquired every 60 s (λex 492 nm; λem 518 nm). In the truncated ssDNA activator assay, full-length ssDNA samples (positive control, P_Ctrl) were normalized to 1-fold fluorescence signal, and the fluorescence signals of other truncated activators were calculated by taking the ratio to the fluorescence signal of the positive group (33). Meanwhile, RNase-free water served as the negative control (N_Ctrl).

For two segment combinations as an activator assay, the Cas12a/crRNA complex was pre-incubated for 5 min at 37°C through mixing a final concentration of 50 nM LbCas12a, 50 nM crRNA, 50 nM different hairpin DNA, 50 nM 3′(x) segment or/and 5′(y) segment. The reaction was initiated by adding the final concentration of 200 nM Probe DNA. Reactions were performed in three replicates (20 μl) at 37°C for 2 h and fluorescence measurements were acquired every 60 s (λ_ex_ 492 nm; λ_em_ 518 nm). Full-length ssDNA served as the positive control (P_Ctrl, 100% activation effect), while RNase-free water served as the negative control (N_Ctrl) to activate Cas12a.

For cleavage of hairpin DNA assay, the Cas12a/crRNA complex was pre-incubated for 5 min at 37°C through mixing a final concentration of 50 nM LbCas12a, 50 nM crRNA and 1 nM full-length ssDNA activator. The reaction was initiated by adding the final concentration of 200 nM different fluorescently labeled hairpin DNA reporters. Reactions were performed in three replicates (20 μl) at 37°C for 2 h and fluorescence measurements were acquired every 60 s (λ_ex_ 492 nm; λ_em_ 518 nm). Probe DNA served as the control.

### High-resolution denaturing polyacrylamide gel electrophoresis (PAGE) assays

High-resolution denaturing PAGE experiments were obtained with 20% polyacrylamide in TBE and 8 M urea buffer. Gel solution was prepared by mixing 24 g urea, 5 ml 10 × TBE buffer, 25 ml 40% acrylamide/bisacrylamide solution, 19 ml ddH_2_O, 166 μl 30% APS and 20 μl TEMED. Five synthetic LNA-modified DNA Ladder was used as the ssDNA standard.

For gel analysis of LNA-modified ssDNA reporters, the Cas12a/crRNA complex was pre-incubated for 5 min at 37°C through mixing a final concentration of 50 nM LbCas12a, 50 nM crRNA and 20 nM full-length activator. The reaction was initiated by adding final concentrations of 500 nM LNA-modified ssDNA reporter. Reactions were performed in four replicates (20 μl) at 37°C and stopped at different times (30 min, 1 h, 2 h, 4 h, 8 h, 12 h and 18 h) by heating the solutions for 10 min at 65°C to inactivate the Cas12a enzyme. The cleaved samples were then mixed with 2 × TBE–urea sample buffer and denaturing PAGE gel was performed at room temperature, at a constant voltage of 400 V for about 4 h. Gel was scanned by an Invitrogen iBright imaging system (Invitrogen, Singapore) with its built-in FAM channel, and the intensities of the bands were quantified with ImageJ software (National Institutes of Health, USA).

For gel analysis of different hairpin DNA reporters, the Cas12a/crRNA complex was pre-incubated for 5 min at 37°C through mixing a final concentration of 50 nM LbCas12a, 50 nM crRNA and 1 nM full-length activator. The reaction was initiated by adding final concentrations of 200 nM different hairpin DNA reporters. Reactions were performed in six replicates (20 μl) at 37°C and stopped at different times (15 min, 30 min, 1 h, 2 h, 4 h, 12 h) by heating the solutions for 10 min at 65°C to inactivate the Cas12a enzyme. The PAGE analysis procedure was consistent with samples in LNA-modified ssDNA reporters assay.

### Cell culture

All cells were cultured in a humidified atmosphere of 5% CO_2_ at 37°C. Two cell lines (MCF-7 and Hela) were cultured in a MEM media supplemented with 10% FBS, while cell line HEK293T was cultured in a high-glucose DMEM media. The cell-free DNA (cfDNA) of HEK293T, Hela and MCF-7 cells (5 × 10^5^/ml, 6 × 10^5^/ml and 7 × 10^5^/ml) were extracted using the Ezup cfDNA Extraction Kit (34) (Sangon Biotech Co. Ltd, China) according to the manufacturer's instruction. The extracted cfDNA was routinely stored at −80°C before incubation with our detection system.

### Evaluations with synthesized gene

The gene used in this work to evaluate the analytical performance of our amplification-free Cas12a method was synthesized by Sangon Biotech Co. Ltd (Shanghai, China) and then cloned into a PUC-57 vector. For fluorescence measurement using the CALSA method, the Cas12a/crRNA complex was pre-incubated for 5 min at 37°C through mixing a final concentration of 50 nM LbCas12a, 50 nM crRNA1, 50 nM Hairpin0 and 200 nM LNA-ss4. The reaction was initiated by adding different final concentrations of the target gene (0 fM, 10 fM, 50 fM, 100 fM, 500 fM, 2 pM, 10 pM, 40 pM, 100 pM). Reactions were performed in three replicates (20 μl) at 37°C for 2 h and fluorescence measurements were acquired every 60 s (λ_ex_ 492 nm; λ_em_ 518 nm). For fluorescence measurement with the direct Cas12a detection method, the Cas12a/crRNA complex was pre-incubated without Hairpin0 and LNA-ss4, and the reaction was initiated by adding different final concentrations of target gene (0, 2, 10, 40, 800 pM). The fluorescence measurements process was consistent with the CALSA method. In the specificity test assay, the wild-type gene served as the positive control (1-fold fluorescence signal), while RNase-free water served as the negative control to activate Cas12a. The fluorescence signals of other mutant genes (100 fM in the CALSA method and 100 pM in the direct Cas12a detection method) were calculated by taking the ratio to the fluorescence signal of the positive group (33). The limit of detection (LoD) was calculated as 3.3σ/*S* (σ is the standard deviation and *S* is the slope of the calibration curve). In the quantitative real-time PCR (qPCR) experiments assay, the plasmid was diluted into various final concentrations ranging from 16.5 to 1.65 aM. The samples were subjected to real-time PCR using iTaq SYBR Green Supermix (Bio-Rad, USA) and the CFX Connect Real-Time System (Bio-Rad, USA). The primers were 5′-GCTTCAGCGTTCTTCGGAATG-3′ (forward) and 5′-GCGGTAAGGCTTGAGTTTC-3′ (reverse).

### One-pot cfDNA detection from different cell lines

Synthetic BRCA-1 at concentrations ranging from 10 fM to 100 pM were added to the CALSA system to make a calibration curve. In the CALSA reaction system, the Cas12a/crRNA complex was pre-incubated for 5 min at 37°C through mixing a final concentration of 50 nM LbCas12a, 50 nM crRNA1, 50 nM crRNA2, 50 nM Hairpin0 and 200 nM LNA-ss4. Reactions were then performed in three replicates (20 μl) at 37°C for 2 h and fluorescence measurements were acquired every 60 s (λ_ex_ 492 nm; λ_em_ 518 nm). For the cfDNA detection, total extracted cfDNA samples from different cell lines (each reaction was performed in six replicates) were added to the CALSA reaction system (refers to the procedure of synthetic BRCA-1 detection) to initiate the reaction. The statistical test was performed using a two-way ANOVA test with Tukey's multiple comparison test (35) in the software of GraphPad Prism 8 (Version 8.2.1), where ns refers to not significant with *P* > 0.05, and the asterisks denote significant differences with *P* < 0.0001 (***), *P* < 0.001 (**), *P* < 0.01 (*). For the detection of synthetic BRCA-1 and cfDNA by direct Cas12a detection method, the Cas12a/crRNA complex was pre-incubated without Hairpin0 and LNA-ss4, instead by 200 nM Probe DNA. The reaction was initiated by adding different final concentrations of the target gene (0, 0.1, 1, 50, 100, 200, 300, 400, 500 pM) and extracted cfDNA samples. The fluorescence measurements process was consistent with the CALSA method.

## Results

### Tolerance of Cas12a to truncated ssDNA activators for *trans*-cleavage activity

As reported, recognition of genes by CRISPR/Cas12a relies on two crucial steps, involving both the PI domain's scanning of the PAM sequence within the target gene and the complementary binding of the 3′ end of crRNA to the target strand (TS) of the gene (36,37). Previous studies have demonstrated that the CRISPR/Cas12a system can recognize both dsDNA and ssDNA under the guidance of crRNA (38). Moreover, the recognition of ssDNA does not strictly rely on the presence of PAM sequences and the truncated ssDNA affects the triggering of Cas12a's *trans*-cleavage activity (25,35,38,39). To further explore whether the sequence composition of truncated ssDNA and the length of crRNA spacer affected the *trans*-cleavage activity of Cas12a, we designed three diverse crRNAs referred to as ‘N’, ‘E’ and ‘P’, and an array of complementary truncated ssDNA. The ‘N’ crRNA and ‘E’ crRNA contained 20-nt of spacers but differed in base compositions (sequences provided in Supplementary Table S1 and S2). The ‘P’ crRNA contained a longer spacer (25 nt) than the ‘N’ and ‘E’ crRNAs (Supplementary Table S3). Additionally, a fluorescent probe DNA containing a TA-rich sequence (Supplementary Table S4), which is known for its pronounced cleavage efficiency (13), was utilized to validate the *trans*-cleavage activity of Cas12a.

**Figure 1.**
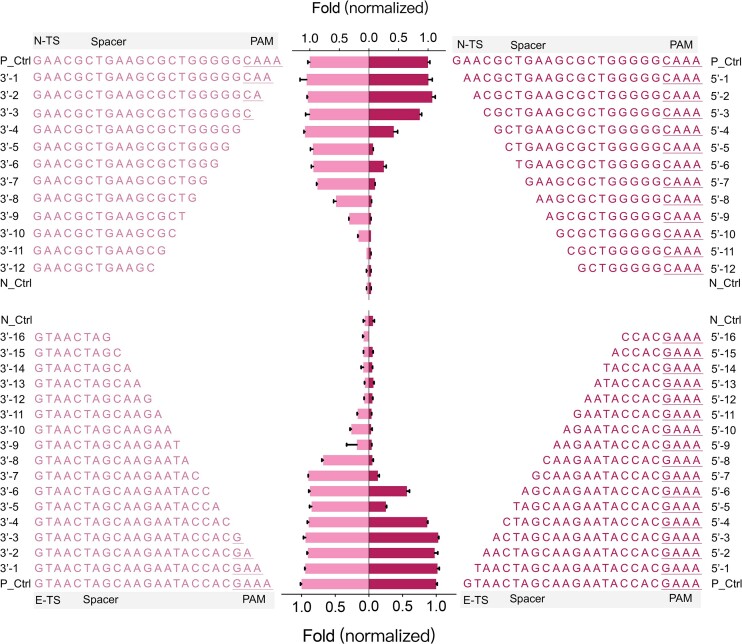
Tolerance of LbCas12a to the truncated ssDNA from both 5′ and 3′ ends of N-TS (up) and E-TS (low) for *trans*-cleavage activity. Full-length ssDNA samples (positive control, P_Ctrl) were normalized to 1-fold fluorescence signal, and the fluorescence signals of other truncated activators were calculated by taking the ratio to the fluorescence signal of the positive group. Meanwhile, RNase-free water served as the negative control (N_Ctrl). The underline marks the sequence in the PAM region. Data are expressed as mean ± SD (*n* = 3).

As depicted in Figure 1 and Supplementary Figure S1, we investigated the impact of truncating ssDNA from both the 5′ and 3′ ends by one base step. The results showed that truncating the first 7 bases from the 3′ end of the ssDNA did not significantly affect the activation effect for ‘N’ crRNA and ‘E’ crRNA on LbCas12a. This result corroborated the earlier study that PAM-less ssDNA can trigger the *trans*-activity of Cas12a (18). For both ‘N’ and ‘E’ crRNAs, truncation of the 8th base or more bases from the 3′ end resulted in a significant reduction and complete abolishment of the ssDNA’s ability to activate LbCas12a. Meanwhile, the activation effect of the truncated ssDNA of ‘N’ and ‘E’ crRNAs both gradually diminished when shortening the 5th base or more bases from the 5′ end. These indicated that truncated ssDNA triggering a decreased *trans*-activity of LbCas12a may not be influenced by the base compositions of truncated ssDNA. Furthermore, the difference in the activating LbCas12a by different truncated numbers of bases between 3′ and 5′ ends was attributed to the presence of the PAM region in 3′ ends. Therefore, excluding the effect of PAM (4 nt) in the 3′ ends, the LbCas12a's *trans*-cleavage activity towards 16-nt ssDNA significantly reduced, and completely lost towards 13-nt ssDNA. These results were consistent with previous reports that ssDNA with more than 14-nt bases is necessary to interact with crRNA and initiate *trans*-cleavage activity of Cas12a (31,39). The ‘P’ system with longer spacer and complement truncated ssDNA were used to trigger the *trans*-activity of FnCas12a and AsCas12a (Supplementary Figure S2 and S3). Similarly, FnCas12a showed reduced *trans*-cleavage activity when ssDNA was less than or equal to 16 nt (excluding the length of PAM), while AsCas12a showed a detectable activity with the truncated activator in 14-nt length. This suggested that the tolerance of the truncated activator was not significantly influenced by crRNA spacer length. Therefore, these results indicated that the *trans*-cleavage activity was sensitive to truncations of the ssDNA activators across the tested orthologs and the minimum length of activators tolerated by Cas12a orthologs was 14 nt. Moreover, the tolerance appeared to not be influenced by the sequence composition of truncated ssDNA and the length of the crRNA spacer. This result provided a theoretical foundation for designing nucleic acid elements in subsequent signal amplification methods in this study.

### Switching Cas12a activity through combinations of two truncated ssDNA segments

**Figure 2.**
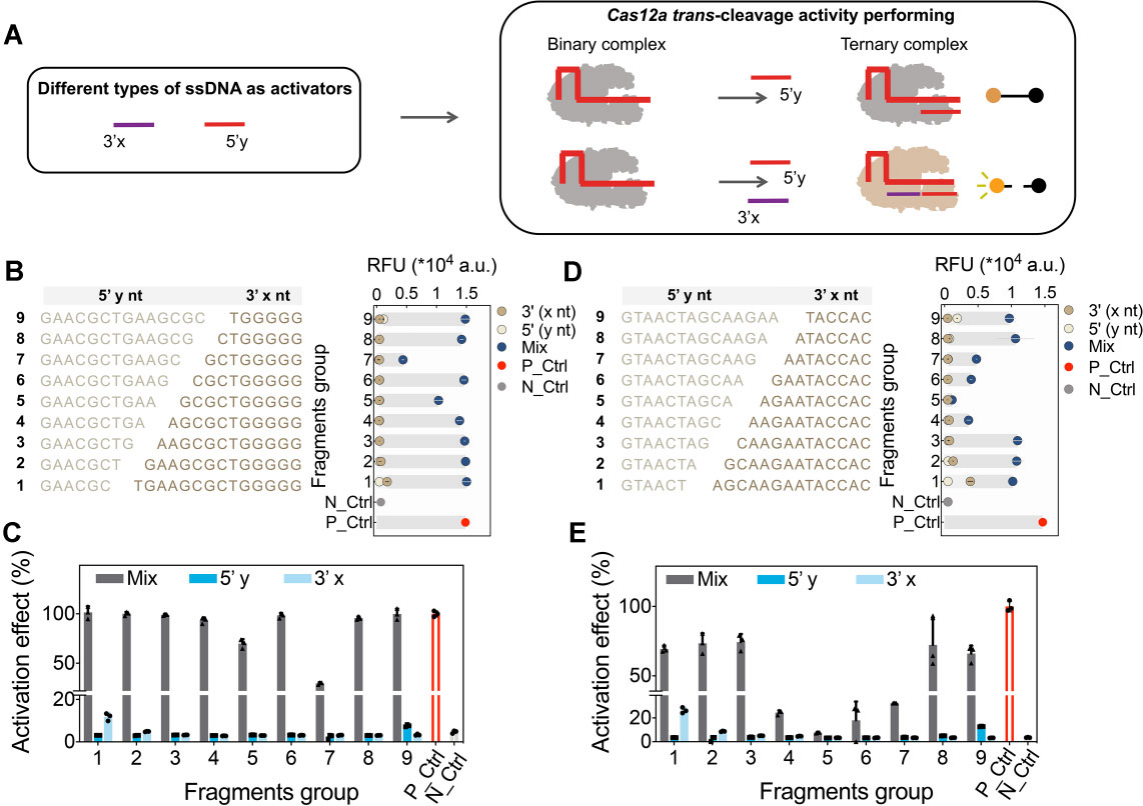
Switching LbCas12a *trans*-cleavage activity via combinations of two truncated ssDNA segments. (**A**) Schematic diagram of the combination of two ssDNA segments as activators to activate LbCas12a *trans*-cleavage activity. (**B**) The sequences of 5′(y) and 3′(x) for ‘N’ crRNA and their activation effects on LbCas12a, both individually and in combination. (**C**) The activation effect of 5′(y) and 3′(x) alone as well as the combination of the two segments for ‘N’ crRNA on LbCas12a. (**D**) The sequences of 5′(y) and 3′(x) for ‘E’ crRNA and their activation effects on LbCas12a, both individually and in combination. (**E**) The activation effect of 5′(y) and 3′(x) alone as well as the combination of the two segments for ‘E’ crRNA on LbCas12a. Full-length ssDNA served as the positive control (P_Ctrl, 100% activation effect), while RNase-free water served as the negative control (N_Ctrl) to activate Cas12a. Data are expressed as mean ± SD (*n* = 3).

We used the complementation experiments to explore whether the combination of two ssDNA segments, 3′(x) and 5′(y) could together activate the *trans*-cleavage activity of LbCas12a. In the experimental setup, the missing segment was added to the reaction system, where only individual 5′(y) or 3′(x) segments were initially present (Figure 2A). For this, we designed an array of 5′(y) segments ranging from 6-nt to 14-nt, and corresponding 3′(x) segments ranging from 14 nt to 6 nt. Each could bind to the different regions of the 20-nt spacer of ‘N’ crRNA (Figure 2B). The results showed that the individual 5′(y) segment and 3′(x) segment were unable to effectively activate LbCas12a, which aligned with previous results that truncated activators shorter than 14-nt cannot active LbCas12a (31,39). However, the segment combination was able to efficiently reactivate the *trans*-cleavage activity of LbCas12a, with some combinations showing lower than or equal to the activity of the full-length activator control (Figure 2C). Next, we also designed an array of 5′(y) segments and corresponding 3′(x) segments to validate the activation activity of combination segments for ‘E’ crRNA (20-nt spacer) and ‘P’ crRNA (Figure 2D and Supplementary Figure S4). The segment combinations from ‘E’ crRNA showed relatively low activation efficiency on LbCas12a, which was generally lower than the 75% activation effect (Figure 2E). We thought the difference in re-activation efficiency by the segment combination between ‘N’ crRNA and ‘E’ crRNA could be attributed to the sequence compositions. For ‘P’ crRNA, the length of 5′(y) segments ranged from 15-nt to 22-nt, and corresponding 3′(x) segments ranged from 10 nt to 3 nt. Each could bind to the different regions of the 25-nt spacer. Similar to the ‘E’ crRNA, the segment combinations of ‘P’ crRNA also presented low activation efficiency on LbCas12a (Supplementary Figure S5). However, the segment combinations of ‘P’ crRNA and individual 5′(y) segments longer than or equal to 19-nt in length showed obvious activation efficiency on AsCas12a and FnCas12a (Supplementary Figure S6).

The recognition and activation process of Cas12a by the target gene primarily rely on the complementary pairing between crRNA and TS ssDNA, mediated by the PI domain of the Cas12a protein, which induces protein conformational rearrangement (31,37). Next, we analyzed possible factors to affect the activation efficacy of segments combination, including melting temperatures (Tm), GC content and the number of base pairings with crRNA of the segments (40–42). Firstly, we analyzed Tm values of 5′(y) segments and 3′(x) segments of ‘N’ crRNA and ‘E’ crRNA, both of which had spacer in 20-nt length. The results showed that when the Tm value of one of the 5′(y) segments or 3′(x) segments in the combinations were above 37°C, the combination exhibited relatively high activation activity (Supplementary Figure S7). This might be because these fragments were able to bind to crRNA and form a transitorily stable secondary structure at 37°C (31,43,44). Then, we used ‘N’ and ‘E’ crRNA as examples to design different crRNAs and 5′(y) as well as 3′(x) activators consisting of GC content ranging from 40 to 70% (Supplementary Table S5-S10). The results showed that the increase of GC contents only slightly improved the activation efficacy of the 5′(y) segment or 3′(x) segment with initial Tm higher than 37°C (Supplementary Figures S8–S11). Additionally, the GC contents seem to have less effect on the activation efficiency of the segment combinations.

To investigate the impact of the number of base pairings, we extended the spacer of ‘N’ crRNA to 25-nt and shortened the spacer of ‘P’ crRNA to 20-nt, respectively (Supplementary Tables S11 and S12). For extended ‘N’ crRNA, the activation efficiency of most segment combinations decreased when the number of base pairings was increased for 5′(y) segments (Supplementary Figure S12). Meanwhile, shortening the base pairing number of 5′(y) segments for ‘P’ crRNA would slightly enhance the activation efficacy of some segment combinations (Supplementary Figure S13). These results were consistent with previous structural data that Cas12a orthologs do not utilize region after the 20th base to guide RNA for target recognition (45,46). It is likely that the longer 3′end sterically inhibits trans DNA interacting with the RuvC domain (42,47).

### Controlling Cas12a activity through structural variations of two activator segments

Based on the findings showcasing the activation potential of segment combinations on LbCas12a, we formulated a hypothesis that one of these truncated ssDNA segments could function as a ‘switch’ to regulate LbCas12a activation in the development of novel signal amplification methods. To this end, we identified two truncated ssDNA segments from group 2 of the ‘N’ system, which exhibited favorable characteristics such as a high signal-to-noise ratio (SNR) and low background signal. These segments were selected to serve as nucleic acid elements in the signal amplification method. To optimize their performance, structural modifications were implemented on these two ssDNA segments. Previous studies have indicated that activated LbCas12a can degrade hairpin DNA with a large loop or 3′ overhang (48,49). We conjectured whether hairpin DNA with a small loop and blunt terminal could mitigate degradation by the activated LbCas12a within the reaction system. We tested the cleavage efficiency of six hairpin DNAs with/without a 3′ overhang, diverse loop sizes, and different stem lengths. The results demonstrated that H0 with a small loop, blunt terminal and long stem was slightly cleaved by the activated LbCas12a within 2 h (sequences provided in Supplementary Table S13, Supplementary Figures S14 and S15). Hence, one of the ssDNA segments was designed to hairpin structure of H0.

**Figure 3.**
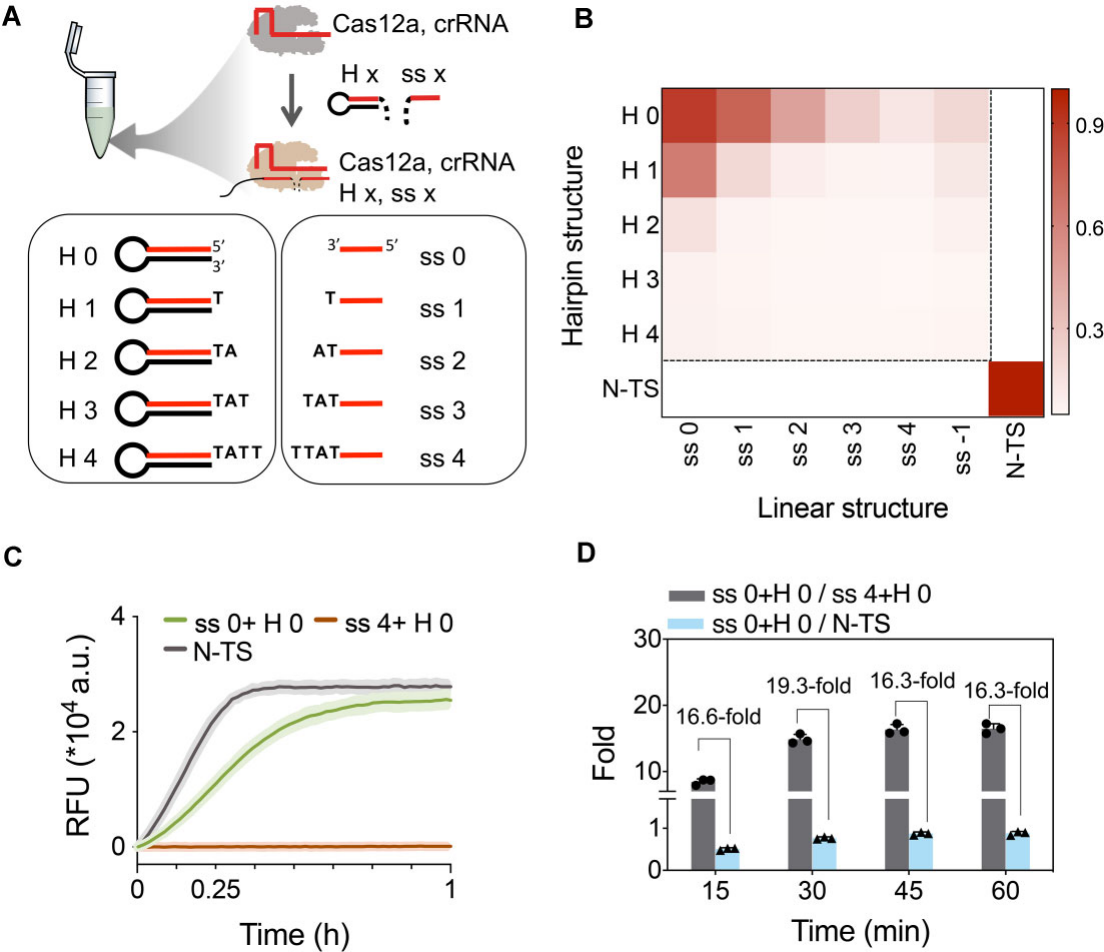
Controlling LbCas12a activity via structural variations of two activator segments. (**A**) Schematic illustration of the structures of Hairpin DNA and straight ssDNA (Hx and ssx), as well as the procedure for activating LbCas12a through the combinations of Hx and ssx. (**B**) Heat map illustrating the activity of H0-4 and ss0-4 from combination 2 of N-TS in activating LbCas12a *trans*-cleavage activity. (**C**) Time-dependent fluorescence signals of ss0 + H0, ss4 + H0 and N-TS in activating LbCas12a *trans*-cleavage activity. (**D**) The differential activation of LbCas12a *trans*-cleavage activity by ss0 + H0 compared to ss4 + H0 and N-TS at various time intervals, respectively. Data are expressed as mean ± SD (*n* = 3).

Additionally, TA-rich sequences of varying lengths were appended at the terminal of the ‘x’ and ‘y’ segments to introduce ‘steric hindrance effects’, which might prevent the allosteric unblocking of the catalytic site and lead to the inactivation of the *trans*-cleavage activity of Cas12a at the beginning of the reaction (39). In our principle design of autocatalysis amplification strategy, the ssDNA segments with or without TA-rich extension would play a ‘switch’ role to control the activation of Cas12a. Specifically, the ssDNA segments with a TA-rich extension could not activate Cas12a, but when the TA-rich extension was cut off, the segment combinations could efficiently activate Cas12a and further trigger cascade amplification signals through positive feedback. Therefore, we designed different constructs of Hairpin0-4 (H0-4) and ss0-4, respectively (Figure 3A). Through a pairwise crossover verification approach, we assessed the activation effects of 25 combinations. The results revealed that the combination of H0 with ss0 yielded the most robust activation effect, with activation gradually diminishing as the number of introduced bases at the 3′ end of ss0 and 5′ end of H0 increased (Figure 3B). Conversely, other combinations did not exhibit significant activation effects on LbCas12a. Notably, when ss4 was substituted with ss0 in combination with H0, rapid activation of LbCas12a and subsequent production of *trans*-cleavage activity were observed within a short duration (Figure 3C and Supplementary Figure S16). Furthermore, we analyzed the SNR of this ‘switch’ element at various time points. As depicted in Figure 3d, the effective SNR reached 16.6 times at 15 min, with a maximum SNR of 19.3 times at 30 min. Significantly, the activation effect achieved by the ss0 and H0 combination was comparable to that of complete N-TS ssDNA. These results emphasized the substantial potential of ss4 as a pivotal ‘switch’ element in constructing LbCas12a-based signal amplification methods when combined with H0.

### The site-directed regulation of LbCas12a cleavage activities by LNA substitutions

Previous research has suggested that the RuvC domain, a nuclease found in several CRISPR-associated proteins, may be responsible for both the *trans* and *cis* activities of Cas12a (37,50). Therefore, we intended to employ the specific modifications against nuclease to enable the site-directed cleavage of the ‘switch’ ssDNA element ss4 into ss0 by the *trans*-cleavage activity of LbCas12a. Ultimately, developing an idealized Cas12a autocatalysis amplification method driven by the ‘switch’ elements ss0 and H0. We firstly tested three common modifications including 2′-*O* methyl (2′-OMe), Phosphorothioate (PS) and LNA modifications (Supplementary Figure S17a) (51–53). Through the fluorescence measurement, we found that the all-site modification of 2′-OMe and PS could only provide partial protection to the reporter ssDNA from degradation, while only the full LNA modifications (LNA1) could completely shield the reporter from the *trans*-cleavage activity of LbCas12a over the 150-minute duration (Supplementary Figures S17b, S17c, S18a–S18c). Moreover, it could be observed that LNA2 and LNA3 were slightly *trans*-cleaved by LbCas12a, while LNA4 and LNA5 were almost completely degraded by LbCas12a with a sharp increase in fluorescence intensity. These results exhibited a gradient resistance effect against the *trans*-cleavage activity of LbCas12a from LNA1 to LNA5 (Supplementary Figure S18c). We further employed high-resolution PAGE to accurately characterize and analyze the resistance effect against the *trans*-cleavage activity induced by LNA modifications. PAGE gel results also demonstrated stable substrate bands for LNA1 when extending the reaction time to 18 h (Supplementary Figure S18d and S18e). Meanwhile, product bands of LNA2 appeared only after extending the reaction time to 10 h, and LNA3 displayed product bands after 4 h. Additionally, the PAGE gel analysis not only demonstrated the consistent cleavage efficiency on LNA4 and LNA5 of 51% and 61% at the 2-hour timepoint with the results obtained from the fluorescence assay, but also strikingly found that the cleavage product fragment of LNA5 appeared slightly higher than that of LNA4 (Supplementary Figures S18d, S18f). We supposed this discrepancy in the band positions of the cleavage products of LNA4 and LNA5 might be attributed to the directional nature of LbCas12a's *trans*-cleavage activity, which randomly cleaves free ssDNA from the 3′ end to the 5′ end. This finding was also reported by Wei's group at the time of the present work's submission (49). Consequently, the cleavage product of LNA5 consisted of a 5-nt TTATT (5′-3′) fragment, while the cleavage product of LNA4 comprised a 4-nt TTAT (5′-3′) fragment. This observation suggested that the cleavage site of *trans*-cleavage activity could be regulated by modifying the positions of LNAs.

**Figure 4.**
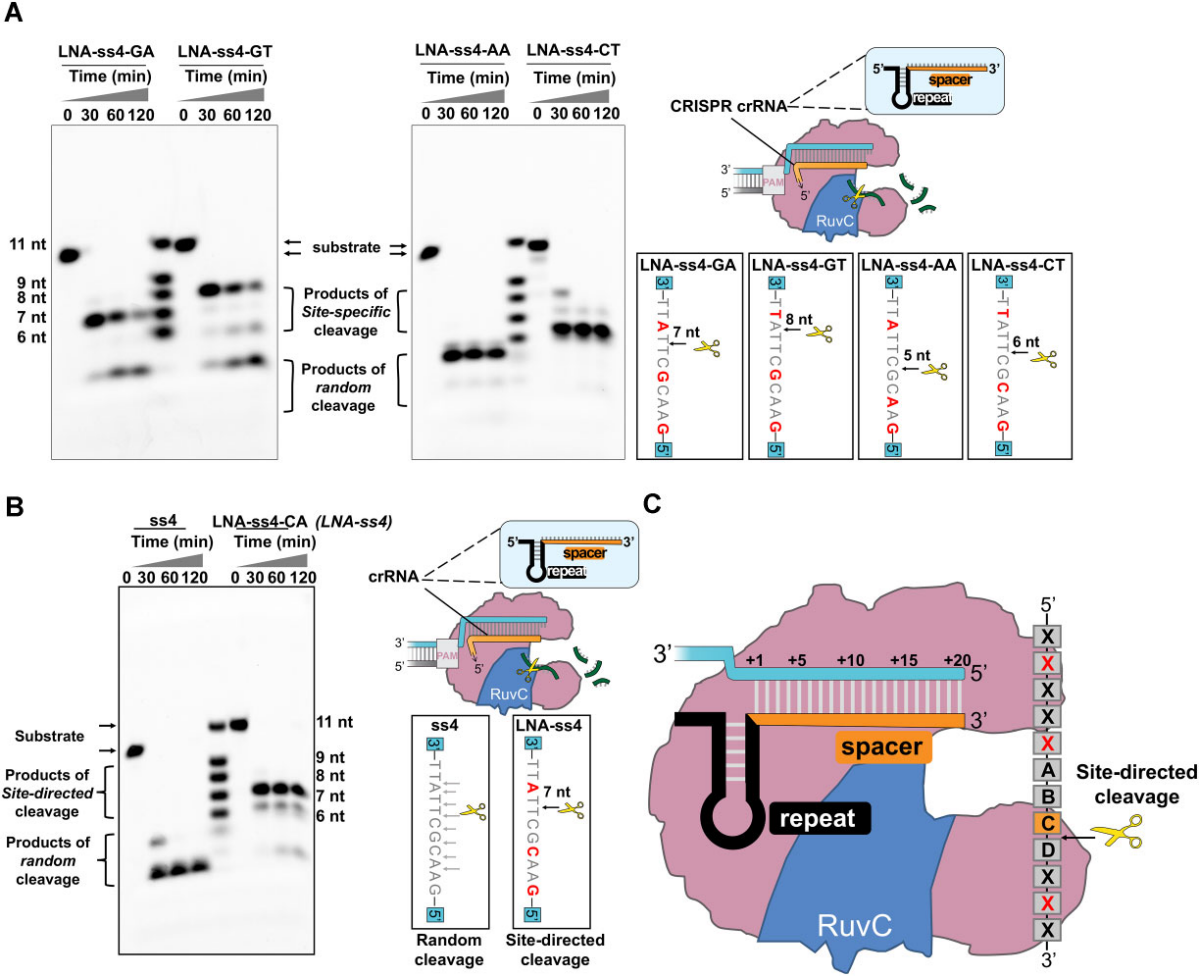
High-resolution PAGE analysis of the LbCas12a *trans*-cleavage reaction on LNA-modified ss4 at different time intervals. (**A**) High-resolution PAGE analysis and schematic illustration of the LbCas12a *trans*-cleavage on LNA-ss4-GA, LNA-ss4-GT, LNA-ss4-AA and LNA-ss4-CT. (**B**) High-resolution PAGE analysis and schematic illustration of the LbCas12a *trans*-cleavage on wild type ss 4 and LNA-ss4-CA. (**C**) The model of site-directed regulation by LNA substitutions where red color shows LNA modified bases.

To further validate the speculation and achieve precise site-specific cleavage using LNA modifications, we designed multiple groups of probes with different positions and numbers of LNA modifications (Supplementary Table S4). We employed high-resolution PAGE and a synthetic ssDNA marker to investigate the regulatory effect of LNA modifications on LbCas12a's *trans*-nuclease activity. As depicted in Figure 4A, upon full activation of LbCas12a protein, it efficiently cleaved the LNA-modified substrate LNA-ss4-GA, yielding a specific product of 7 nt in size and random fragments smaller than 6 nt. Over time, the intensity of the specific product bands gradually diminished while the random fragments increased. However, when the LNA modification site was shifted from the third position to the second position near the 3′ end (LNA-ss4-GT), the size of the specific product increased to 8 nt. Moreover, as the reaction time extended, the specific product bands weakened, and the random fragments increased, while the cleavage position remained consistent. The observed change in the specific cleavage products between LNA-ss4-GA and LNA-ss4-GT confirmed the tendency of LbCas12a's *trans*-cleavage activity to cleave from the 3′ end to the 5′ end of ssDNA, demonstrating the feasibility of shifting LbCas12a's random cleavage towards site-directed cleavage by introducing LNA modifications at different sites.

To further substantiate that LNA modifications could regulate LbCas12a to achieve site-directed cleavage and that the regulatory effect on LbCas12a's *trans*-nuclease activity was stable within a limited reaction time, we changed LNA-modification sites on the ss4 segment. When the LNA-modification site at the 5′ end of LNA-ss4-GA was changed from the fifth position G to the third position A (LNA-ss4-AA), we speculated that the product would primarily consist of 5 nt GAACG (indicated by the gray arrow in Figure 4A), and high-resolution PAGE confirmed that the cleavage product indeed comprised fragments smaller than 6 nt. Similarly, when the LNA-modification site at the 5′ end of LNA-ss4-GT was changed from the fifth position G to the fourth position C (LNA-ss4-CT), as shown in Figure 4A, the LNA-ss4-CT probe exhibited 8 nt and 7 nt bands at the 30-min reaction time point. At the 1-h and 2-h time points, the 8 nt band disappeared with more than 90% of the products being 6 nt fragments, and there were no random products smaller than 6 nt. These experimental results aligned with our speculation. Moreover, when we changed the LNA-modification site at the 3′ end of LNA-ss4-CT from T to the adjacent A (LNA-ss4-CA, Figure 4B), the specific product shifted from 6 nt to 7 nt. These findings demonstrated that the LNA modifications effectively regulated LbCas12a's *trans*-cleavage activity, converting its random degradation of ssDNA into site-directed cleavage, and enabling precise cleavage of the ‘switch’ element LNA-ss4 into ss0.

Based on these results, we proposed a model to explain this pattern, wherein the introduction of LNA modifications at three specific sites on a 12 nt ssDNA restricted the cleavage site of LbCas12a's *trans*-nuclease activity to a precise location (as indicated by the dotted line between c and d in Figure 4C). As a result, the 12 nt substrate undergoes cleavage into two fragments, measuring 8 nt and 4 nt in size. This model not only could be used in this work to construct a high-performance signal amplification method, but also provided more flexible options for CRISPR/Cas12a or nuclease-based biosensing and logic circuit applications.

### A LbCas12a autocatalysis amplification effect for DNA sensing

**Figure 5.**
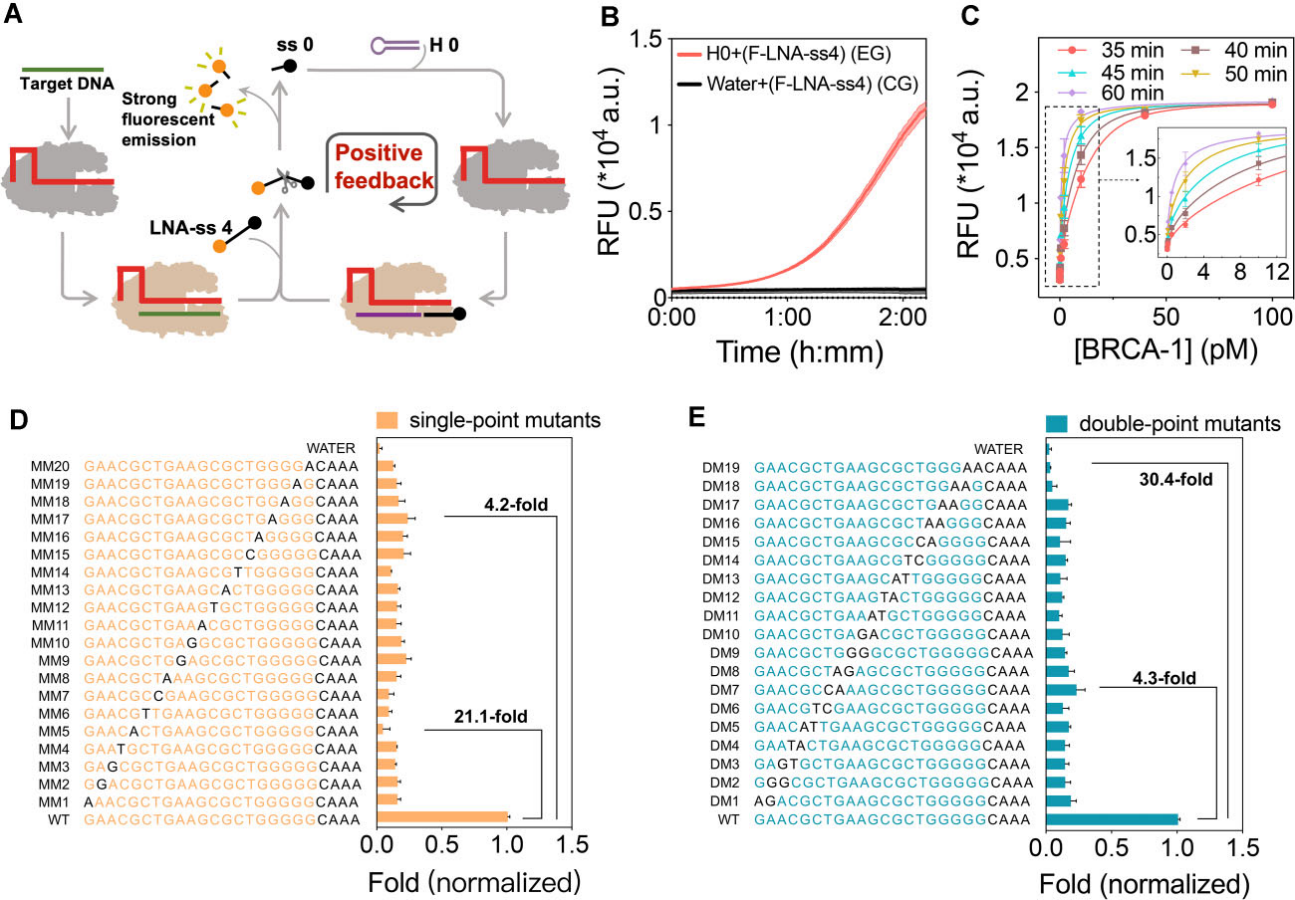
Highly sensitive and specific detection of DNA by CALSA. (**A**) Principle of CALSA: utilizing a LbCas12a autocatalysis-driven positive feedback circuit for exponentially amplified DNA detection. (**B**) The contribution of a positive feedback loop driven by H0 and F-LNA-ss4 in the CALSA method (target plasmid: 50 fM). EG represented the experiment group with H0 and LNA-ss4, CG represented the control group without H0. (**C**) Experimental analysis of time-dependent fluorescence signal changes in CALSA in response to target plasmid at various time intervals. (**D**, **E**) Bar graphs showing the relative fluorescence fold change of the MM1-MM20 and DM1–DM19 mutant DNA activators compared to the WT activator. Data are expressed as mean ± SD (*n* = 3).

Building upon the LNA-ss4 modification, we have developed a novel positive feedback signal amplification strategy and an amplification-free gene detection method termed CALSA (Figure 5A). CALSA utilized H0 and fluorescently labeled LNA-ss4 as (F-LNA-ss4) essential signal amplification elements, initiating an autocatalytic reaction mediated by LbCas12a. As a ‘one-pot’ system, the crRNA-Cas12a complex remained inactive, and both H0 and F-LNA-ss4 failed to activate LbCas12a in the absence of the target gene. Subsequently, the presence of even trace amounts of the target gene triggered the recognition and binding of the crRNA-Cas12a complex to the target gene, leading to the activation of LbCas12a's *trans*-cleavage activity. Consequently, free F-LNA-ss4 underwent site-directed cleavage by LbCas12a and generated ss0. The resulting ss0 then combined with H0, and further activated LbCas12a to initiate a positive feedback loop. We performed fluorescence measurement experiments to validate that combinations of H0 and F-LNA-ss4 could trigger a positive feedback loop in the CALSA method. As shown in Figure 5B, the experimental groups (EG) in the presence of H0 and F-LNA-ss4 triggered the feedback loop, displaying a significant fluorescence increase. In contrast, the fluorescence signal in control groups (CG) without H0 did not increase. These results demonstrated that combinations of H0 and F-LNA-ss4 could introduce a positive feedback loop in the CALSA method. This feedback loop amplified the signal and activated numerous LbCas12a molecules, resulting in the cleavage of more LNA-ss4 molecules and the generation of a substantial fluorescent signal, enabling the one-pot detection of trace target genes. We further compared the different detection efficiencies using fluorescently Probe DNA and F-LNA-ss4 in the CALSA method, as exhibited in Supplementary Figure S19a, Probe DNA showed a slightly earlier response to the same concentration of plasmid compared with F-LNA-ss4, which was consistent with the different cleavage efficiency of two probes shown in Supplementary Figure 19b. This result implied that both classical fluorescently Probe DNA and F-LNA-ss4 could be used as reporters in the CALSA method.

We further assessed CALSA’s performance by testing its signal amplification efficiency and detection sensitivity using a range of plasmid concentrations. As shown in Figure 5C and Supplementary Figure S20a, CALSA successfully detected target plasmids as low as 50 fM within one hour with clearly distinguishable fluorescence intensities at different concentrations, indicating the effectiveness of CALSA in providing a clear and resolving response to varying target gene concentrations. However, the signal from the 10 fM concentration was indistinguishable from the background, likely due to strong exponential amplification causing background signal interference. Despite this, CALSA outperformed the direct LbCas12a detection method by significantly reducing detection time. Additionally, CALSA demonstrated a three-order of magnitude improvement in detection sensitivity compared to the direct method (1 h for 50 fM versus 1.5 h for 40 pM, Supplementary Figure S20b).

We analyzed the detection results at different time intervals to determine the best detection time for CALSA. Supplementary Figure S21 showed that plasmid concentration and fluorescence values followed calibration curves between 35 and 60 min. Extended detection time improved the fitting quality, with an increase in the R^2^ value from 0.87 (at 35 min) to 0.96 (at 50 min). This indicated that the optimal detection time for CALSA was 50 min. Additionally, by examining the background fluorescence at 50 min, we found that CALSA’s detection limit in this study was around 25 fM. However, the CALSA method could not reach a comparable sensitivity to the gold-standard quantitative real-time PCR (qPCR) method (Supplementary Figure S22), it still exhibited to be a potential isothermal detection tool for point-of-care tests.

We conducted additional experiments to assess CALSA’s specificity by using plasmids with single-point and double-point mutations in the target-binding region (Figure 5D, 5E). Activation efficiency significantly decreased when LbCas12a was activated with mutant plasmids instead of wild-type (WT) plasmids. The decrease ranged from 4.2-fold to 21.1-fold for single-point mutations and from 4.3-fold to 30-fold for double-point mutations. In addition, mutations in the PAM region could also be detected in the CALSA method (Supplementary Figure S23). Furthermore, the specificity of the CALSA method for mutation detection was comparable to the direct Cas12a detection method (Supplementary Figure S24).

### A LbCas12a autocatalysis amplification effect for MCF-7 cell lines sensing

**Figure 6.**
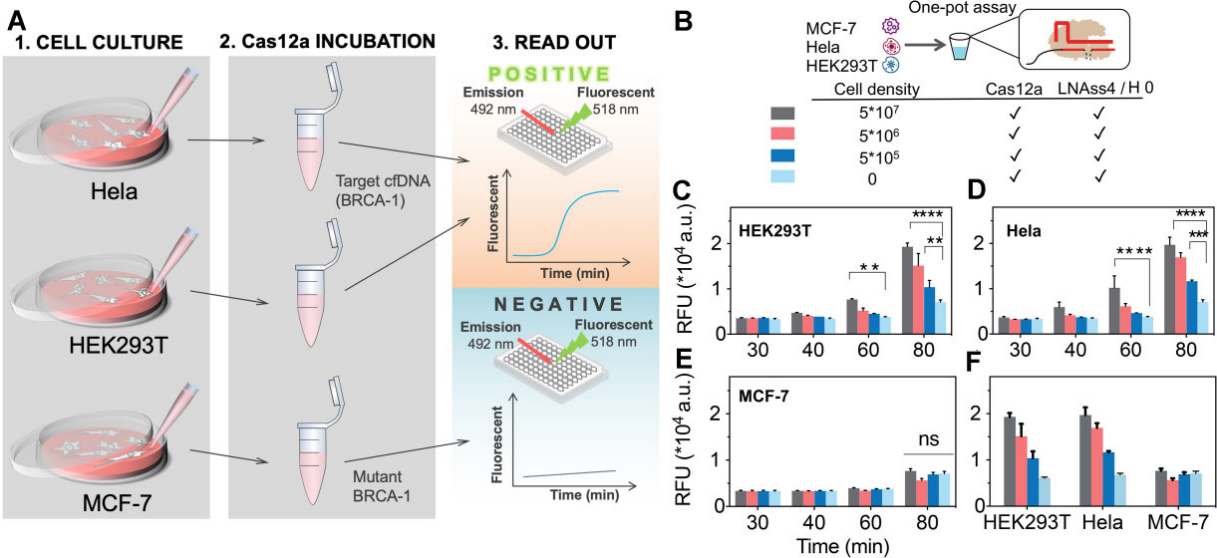
Specific detection of different tumor cell lines by CALSA. (**A**) Schematic diagram depicting the measurement of cell-free DNA (cfDNA) from cell culture media, with HEK293T and Hela cells representing BRCA-1-positive cell lines, while MCF-7 serves as a BRCA-1-negative cell line. (**B**) Schematic diagram illustrating the one-pot detection of different tumor cell lines using CALSA. (**C–E**) Bar graphs illustrating the time-dependent fluorescence changes for HEK293T, Hela, and MCF-7 cell lines at different cell densities. The statistical analysis in (C–E) was performed using a two-way ANOVA test with Tukey's multiple comparison test through GraphPad Prism 8 (Version 8.2.1), where ns refers to not significant with *P*> 0.05, and the asterisks denote significant differences with *P* values < 0.0001 (***), *P* < 0.001 (**), *P* < 0.01 (*). (f) Bar graphs illustrating the fluorescence changes in response to different cell lines at varying cell densities. Data are expressed as mean ± SD (*n* = 6).

Inspired by the impressive sensitivity and mutation specificity achieved with CALSA, we applied it to detect breast cancer through cell-free DNA (cfDNA) analysis, which levels in the blood are typically linked to tumor growth status, as tumors proliferate rapidly (Figure 6A) (54,55). BRCA-1, a cfDNA marker associated with breast cancer (56) and crucial for early breast cancer diagnosis (57), was selected as the target for detection. We validated CALSA’s performance in a serum environment by constructing a calibration curve using various concentrations of the BRCA-1 gene diluted in 1 × PBS and 5% human serum (sequences provided in Supplementary Table S14, Supplementary Figures S25, S26). The results showed consistent fluorescence signals and strong linear correlations (*R*^2^ > 0.9) across different reaction conditions, indicating reliable target DNA measurement. Furthermore, the detection sensitivity of the CALSA method for BRCA-1 was enhanced, with a lower limit of 10 fM and a limit of detection (LOD) of 4.7 fM, which was three orders of magnitude higher than the direct Cas12a detection method (Supplementary Figure S27). These findings suggested that CALSA’s sensitivity can be further enhanced by improving the activation efficiency of different target genes and crRNA. To assess specificity, we detected seven types of ssDNA directly related to breast cancer in the blood. As shown in Supplementary Figure S28, BRCA-1 exhibited significant fluorescence intensity, while non-target DNAs showed similar intensities to the negative signal, even at 100-fold higher concentrations than BRCA-1.

We further tested BRCA-1 in three cell lines, HEK293T, Hela, and MCF-7 by CALSA. MCF-7 cells typically have mutated BRCA-1, while the other cell lines have wild-type BRCA-1. CALSA successfully detected BRCA-1 in HEK293T and Hela cells (Figure 6B–6D and Supplementary Figures S29a, S29b), showing a significant increase in fluorescence intensity with increasing cell density (*P*< 0.01). However, for MCF-7 cells (Figure 6E and Supplementary Figure S29c), the fluorescence signal did not significantly increase with cell density and remained lower than that of HEK293T and Hela cells (Figure 6F). It was attributed that MCF-7 cells were generally known to express mutated BRCA-1 (56,57), which could not target wild-type crRNA to activate LbCas12a in the CALSA method. In contrast, no fluorescent signal of cfDNA was detected in the cell culture samples by the direct Cas12a detection method (Supplementary Figure S30). These results demonstrated CALSA’s ability to quickly screen different tumor cells and analyze their growth density when detecting BRCA-1 in cell culture medium.

## Discussion

CRISPR-Cas systems possess dual functions, providing stringent sequence-based recognition and efficient catalytic cleavage of nucleic acids. This makes them highly promising for signal amplification and biosensing technologies based on the autocatalytic amplification effect driven by a positive feedback loop of nucleic acids. In this study, we presented that the combination of silent split ssDNA could efficiently trigger the Cas12a protein and LNA modifications could mediate random cleavage on ssDNA to site-directed *trans*-cleavage. Through the rational structural and functional design of nucleic acid modules, we build key nucleic acid elements with self-reporting functions in signal amplification circuits, such as H0 and LNA-ss4 in CALSA. By harnessing the efficient positive feedback loop from these two nucleic acid elements, CALSA addressed the limitations of conventional CRISPR-Cas system-based methods that require pre-amplification steps and complex scheme designs. In CALSA, the RNA-guided multiple target recognition of LbCas12a triggered an explosive signal amplification driven by nucleic acid elements. This allowed CALSA to directly process trace amounts of input genomic DNA or ssDNA, such as virus genomic DNA and cancer-associated cfDNA, and produced distinct cascade amplification signals from the LbCas12a autocatalysis-driven positive feedback circuit. Moreover, this LbCas12a system-only signal amplification network in CALSA exhibited extraordinarily high turnover efficiency, enabling faster detection of nucleic acids at a constant physiological temperature compared to other methods powered by the CRISPR-Cas system. Thus, CALSA served as a proof of principle that demonstrated the reconstruction of highly efficient signal amplification strategies capable of one-pot, real-time detection of multiple nucleic acids with ultra-sensitivity and single-base specificity. This development provided a novel paradigm for the advancement of signal amplification circuits with promising potential in molecular diagnosis.

Supplementary Table S15 compared CALSA’s performance with other Cas12a-based nucleic acid detection methods. Although CALSA’s sensitivity did not reach the 5 aM limit achieved by Shi et al.’s self-catalytic amplification system, it outperformed in sensitivity and detection time as it could rapidly detect the target gene at concentrations as low as 25 fM within 50 min. Moreover, the CALSA’s ‘one-pot’ reaction offered several advantages. It avoided the necessity for sophisticated scheme design and the need for extra pre-amplification steps involving multiple enzymes, thermocycling, or initial heating processes to denature dsDNA. Additionally, CALSA generalized the detection of different targets by simply replacing the crRNA, making it simple, easy to operate, and versatile. Importantly, by introducing split ssDNA activators to control the *trans*-activity of Cas12a, CALSA provided greater access to designing a Logic-Gate-based CRISPR/Cas12a biosensing platform, expanding its applicability. However, some limitations should be addressed in the CALSA method. Firstly, the CALSA method could only reach the femtomolar sensitivity. The lower sensitivity than other methods (like CONAN) might attributed to that the designed ‘switch elements’ (H0 and LNA-ss4) were unable to completely silence the activation of Cas12a (Figure 3B and Supplementary Figure S16f). Secondly, the introduction of LNA-modified nucleic acid elements increased the cost of detection by the CALSA method compared with the classic Cas12a assay. Thirdly, the F-LNA-ss4 reporter used in CALSA may not be generally used in other Cas12a detection methods due to the specific sequence and modifications. To address this issue, further optimization of the structure of H0 or the exploration of alternative Cas proteins can be considered to minimize background fluorescence interference.

In summary, CALSA has demonstrated the potential of signal amplification based on the LbCas12a autocatalysis-driven positive feedback circuit in ultrasensitive nucleic acid detection by ingeniously integrating the properties of split ssDNA activators triggering the LbCas12a protein and LNA modifications mediating site-directed *trans*-cleavage. Furthermore, as a proof of concept, CALSA not only paved the way for the development of high-performance signal amplification methods with promising potential in early diagnosis and disease treatment, but also provided a template for various CRISPR-Cas systems. Using split ssDNA-LNA modifications as activators, these systems could precisely control the activation of the CRISPR-Cas system and explore a rich repertoire of innovative applications.

## Supplementary Material

gkae176_Supplemental_File

## Data Availability

The data underlying this article are available in the article and in its online supplementary material. Further raw data supporting the findings of this study except gels are available at https://doi.org/10.6084/m9.figshare.25058459.v1
