# Supplementary material for: An autocatalytic CRISPR-Cas amplification effect propelled by the LNA-modified split activators for DNA sensing
Source: Nucleic Acids Res. 2024 Mar 13;52(7):e39. doi: 10.1093/nar/gkae176 (PMC11040154; doi:10.1093/nar/gkae176)
Supplement: gkae176_Supplemental_File [file gkae176_supplemental_file.pdf]

SUPPLEMENTAL MATERIAL

An autocatalytic CRISPR-Cas amplification effect propelled by the LNA-modified split activators for DNA sensing

Ke Sun[1,2], Lei Pu[1], Chuan Chen[1,3], Mutian Chen[1], Kaiju Li[1], Xinqiong Li[1], Huanqing Li[1] and Jia Geng[1,2]*

[1] Department of Laboratory Medicine, State Key Laboratory of Biotherapy and Clinical Laboratory Medicine Research Center, West China Hospital, Sichuan University, 610041, Chengdu, China

[2] Tianfu Jincheng Laboratory, City of Future Medicine, Chengdu 641400, China

[3] School of Pharmacy, North Sichuan Medical College, 637000, Nanchong, China

* To whom correspondence should be addressed. Tel: +86 028 8695 2279; Email: geng.jia@scu.edu.cn

Supplementary Table S1. Sequences in N-system including crRNA and ssDNA activators.

Name	Sequence# (5'-3')
crRNA1	UAAUUUCUACUAAGUGUAGAUCCCCCAGCGCUUCAGCGUUC
5'-0/3'-0	GAACGCTGAAGCGCTGGGGGCAAA
5'-1	AACGCTGAAGCGCTGGGGGCAAA
5'-2	ACGCTGAAGCGCTGGGGGCAAA
5'-3	CGCTGAAGCGCTGGGGGCAAA
5'-4	GCTGAAGCGCTGGGGGCAAA
5'-5	CTGAAGCGCTGGGGGCAAA
5'-6	TGAAGCGCTGGGGGCAAA
5'-7	GAAGCGCTGGGGGCAAA
5'-8	AAGCGCTGGGGGCAAA
5'-9	AGCGCTGGGGGCAAA
5'-10	GCGCTGGGGGCAAA
5'-11	CGCTGGGGGCAAA
5'-12	GCTGGGGGCAAA
3'-1	GAACGCTGAAGCGCTGGGGGCAA
3'-2	GAACGCTGAAGCGCTGGGGGCA
3'-3	GAACGCTGAAGCGCTGGGGGC
3'-4	GAACGCTGAAGCGCTGGGGG
3'-5	GAACGCTGAAGCGCTGGGG
3'-6	GAACGCTGAAGCGCTGGG
3'-7	GAACGCTGAAGCGCTGG
3'-8	GAACGCTGAAGCGCTG
3'-9	GAACGCTGAAGCGCT
3'-10	GAACGCTGAAGCGC
3'-11	GAACGCTGAAGCG
3'-12	GAACGCTGAAGC
MM1	AAACGCTGAAGCGCTGGGGGCAAA
MM2	GGACGCTGAAGCGCTGGGGGCAAA
MM3	GAGCGCTGAAGCGCTGGGGGCAAA
MM4	GAATGCTGAAGCGCTGGGGGCAAA
MM5	GAACACTGAAGCGCTGGGGGCAAA
MM6	GAACGTTGAAGCGCTGGGGGCAAA
MM7	GAACGCCGAAGCGCTGGGGGCAAA
MM8	GAACGCTAAAGCGCTGGGGGCAAA
MM9	GAACGCTGGAGCGCTGGGGGCAAA
MM10	GAACGCTGAGGCGCTGGGGGCAAA
MM11	GAACGCTGAAACGCTGGGGGCAAA
MM12	GAACGCTGAAGTGCTGGGGGCAAA
MM13	GAACGCTGAAGCACTGGGGGCAAA
MM14	GAACGCTGAAGCGTTGGGGGCAAA
MM15	GAACGCTGAAGCGCCGGGGGCAAA
MM16	GAACGCTGAAGCGCTAGGGGCAAA

MM17	GAACGCTGAAGCGCTGAGGGCAAA
MM18	GAACGCTGAAGCGCTGGAGGCAAA
MM19	GAACGCTGAAGCGCTGGGAGCAAA
MM20	GAACGCTGAAGCGCTGGGGACAAA
WT	GAACGCTGAAGCGCTGGGGGCAAA
DM1	AGACGCTGAAGCGCTGGGGGCAAA
DM2	GGGCGCTGAAGCGCTGGGGGCAAA
DM3	GAGTGCTGAAGCGCTGGGGGCAAA
DM4	GAATACTGAAGCGCTGGGGGCAAA
DM5	GAACATTGAAGCGCTGGGGGCAAA
DM6	GAACGTCGAAGCGCTGGGGGCAAA
DM7	GAACGCCAAAGCGCTGGGGGCAAA
DM8	GAACGCTAGAGCGCTGGGGGCAAA
DM9	GAACGCTGGGGCGCTGGGGGCAAA
DM10	GAACGCTGAGACGCTGGGGGCAAA
DM11	GAACGCTGAAATGCTGGGGGCAAA
DM12	GAACGCTGAAGTACTGGGGGCAAA
DM13	GAACGCTGAAGCATTGGGGGCAAA
DM14	GAACGCTGAAGCGTCGGGGGCAAA
DM15	GAACGCTGAAGCGCCAGGGGCAAA
DM16	GAACGCTGAAGCGCTAAGGGCAAA
DM17	GAACGCTGAAGCGCTGAAGGCAAA
DM18	GAACGCTGAAGCGCTGGAAGCAAA
DM19	GAACGCTGAAGCGCTGGGAACAAA
MM-PAM1	GAACGCTGAAGCGCTGGGGGTAAA
MM-PAM2	GAACGCTGAAGCGCTGGGGGCGAA
MM-PAM3	GAACGCTGAAGCGCTGGGGGCAGA
MM-PAM4	GAACGCTGAAGCGCTGGGGGCAAAG
DM-PAM1	GAACGCTGAAGCGCTGGGGATAAA
DM-PAM2	GAACGCTGAAGCGCTGGGGGTGAA
DM-PAM3	GAACGCTGAAGCGCTGGGGGCGGA
DM-PAM4	GAACGCTGAAGCGCTGGGGGCGAA

[#]Red color indicates mutant sites.

Supplementary Table S2. Sequences in E-system including crRNA and ssDNA activators.

Name	Sequence (5'-3')
crRNA_E	UAAUUUCUACUAAGUGUAGAUGUGGUAUUCUUGCUAGUUAC
5'-0/3'-0	GTAACTAGCAAGAATACCACGAAA
5'-1	TAACTAGCAAGAATACCACGAAA
5'-2	AACTAGCAAGAATACCACGAAA
5'-3	ACTAGCAAGAATACCACGAAA
5'-4	CTAGCAAGAATACCACGAAA
5'-5	TAGCAAGAATACCACGAAA
5'-6	AGCAAGAATACCACGAAA
5'-7	GCAAGAATACCACGAAA
5'-8	CAAGAATACCACGAAA
5'-9	AAGAATACCACGAAA
5'-10	AGAATACCACGAAA
5'-11	GAATACCACGAAA
5'-12	AATACCACGAAA
5'-13	ATACCACGAAA
5'-14	TACCACGAAA
5'-15	ACCACGAAA
3'-1	GTAACTAGCAAGAATACCACGAA
3'-2	GTAACTAGCAAGAATACCACGA
3'-3	GTAACTAGCAAGAATACCACG
3'-4	GTAACTAGCAAGAATACCAC
3'-5	GTAACTAGCAAGAATACCA
3'-6	GTAACTAGCAAGAATACC
3'-7	GTAACTAGCAAGAATAC
3'-8	GTAACTAGCAAGAATA
3'-9	GTAACTAGCAAGAAT
3'-10	GTAACTAGCAAGAA
3'-11	GTAACTAGCAAGA
3'-12	GTAACTAGCAAG
3'-13	GTAACTAGCAA
3'-14	GTAACTAGCA
3'-15	GTAACTAGC
3'-16	GTAACTAG

Supplementary Table S3. Sequences in P-system including crRNA and ssDNA activators.

Name	Sequence (5'-3')
crRNA_P	AAUUUCUACUCUUGUAGAUGCCAGGGACGAAGCGCAAGUGACGGAAU
5'-0/3'-0	CCGTCACTTGCGCTTCGTCCCTGGCGAAA
5'-1	CGTCACTTGCGCTTCGTCCCTGGCGAAA
5'-2	GTCACTTGCGCTTCGTCCCTGGCGAAA
5'-3	TCACTTGCGCTTCGTCCCTGGCGAAA
5'-4	CACTTGCGCTTCGTCCCTGGCGAAA
5'-5	ACTTGCGCTTCGTCCCTGGCGAAA
5'-6	CTTGCGCTTCGTCCCTGGCGAAA
5'-7	TTGCGCTTCGTCCCTGGCGAAA
5'-8	TGCGCTTCGTCCCTGGCGAAA
5'-9	GCGCTTCGTCCCTGGCGAAA
5'-10	CGCTTCGTCCCTGGCGAAA
5'-11	GCTTCGTCCCTGGCGAAA
5'-12	CTTCGTCCCTGGCGAAA
5'-13	TTCGTCCCTGGCGAAA
5'-14	TCGTCCCTGGCGAAA
5'-15	CGTCCCTGGCGAAA
5'-16	GTCCCTGGCGAAA
3'-1	CCGTCACTTGCGCTTCGTCCCTGGCGAA
3'-2	CCGTCACTTGCGCTTCGTCCCTGGCGA
3'-3	CCGTCACTTGCGCTTCGTCCCTGGCG
3'-4	CCGTCACTTGCGCTTCGTCCCTGGC
3'-5	CCGTCACTTGCGCTTCGTCCCTGG
3'-6	CCGTCACTTGCGCTTCGTCCCTG
3'-7	CCGTCACTTGCGCTTCGTCCCT
3'-8	CCGTCACTTGCGCTTCGTCCC
3'-9	CCGTCACTTGCGCTTCGTCC
3'-10	CCGTCACTTGCGCTTCGTC
3'-11	CCGTCACTTGCGCTTCGT
3'-12	CCGTCACTTGCGCTTCG
3'-13	CCGTCACTTGCGCTTC
3'-14	CCGTCACTTGCGCTT
3'-15	CCGTCACTTGCGCT

Supplementary Table S4. Sequences used in the construction of CALSA.

Name	Sequence# (5'-3')
Probe DNA	TTATTATT
Hairpin 0	GAAGCGCTGGGGGCAAATTTTTTTGCCCCCAGCGCTTC
Hairpin 1	TGAAGCGCTGGGGGCAAATTTTTTTGCCCCCAGCGCTTC
Hairpin 2	ATGAAGCGCTGGGGGCAAATTTTTTTGCCCCCAGCGCTTC
Hairpin 3	TATGAAGCGCTGGGGGCAAATTTTTTTGCCCCCAGCGCTTC
Hairpin 4	TTATGAAGCGCTGGGGGCAAATTTTTTTGCCCCCAGCGCTTC
ss 0	GAACGCT
ss 1	GAACGCTT
ss 2	GAACGCTTA
ss 3	GAACGCTTAT
ss 4	GAACGCTTATT
Marker-11 nt	GAACGCTTATT
Marker-9 nt	GAACGCTTA
Marker-8 nt	GAACGCTT
Marker-7 nt	GAACGCT
Marker-6 nt	GAACGC
LNA-ss4	GAACGCTTATT
LNA-ss 4/ LNA-ss4-CA	GAACGCTTATT
LNA-ss4-AA	GAACGCTTATT
LNA-ss4-CT	GAACGCTTATT
LNA-ss4-GA	GAACGCTTATT
LNA-ss4-GT	GAACGCTTATT

#Probe DNA has both 5'FAM and 3'BHQ modifications; Marker DNA and LNA-ss4 (for PAGE) have only 5'FAM modifications; LNA-ss4 have both 5'BHQ and 3'FAM modifications. Red color indicates LNA-modified sites.

Supplementary Table S5. Sequences in N-system (70% GC content) including crRNA and ssDNA activators.

Name	Sequence (5'-3')
crRNA_N_70%	UAAUUUCUACUAAGUGUAGAUCCCCCAGCGCUUCAGCGUUC
WT_N_70%	GAACGCTGAAGCGCTGGGGGCAAA
NO.1 (3'18)_70%	TGAAGCGCTGGGGGCAAA
NO.2 (3'17)_70%	GAAGCGCTGGGGGCAAA
NO.3 (3'16)_70%	AAGCGCTGGGGGCAAA
NO.4 (3'15)_70%	AGCGCTGGGGGCAAA
NO.5 (3'14)_70%	GCGCTGGGGGCAAA
NO.6 (3'13)_70%	CGCTGGGGGCAAA
NO.7 (3'12)_70%	GCTGGGGGCAAA
NO.8 (3'11)_70%	CTGGGGGCAAA
NO.9 (3'10)_70%	TGGGGGCAAA
NO.9 (5'14)_70%	GAACGCTGAAGCGC
NO.8 (5'13)_70%	GAACGCTGAAGCG
NO.7 (5'12)_70%	GAACGCTGAAGC
NO.6 (5'11)_70%	GAACGCTGAAG
NO.5 (5'10)_70%	GAACGCTGAA
NO.4 (5'9)_70%	GAACGCTGA
NO.3 (5'8)_70%	GAACGCTG
NO.2 (5'7)_70%	GAACGCT
NO.1 (5'6)_70%	GAACGC

Supplementary Table S6. Sequences in N-system (60% GC content) including crRNA and ssDNA activators.

Name	Sequence (5'-3')
crRNA_N_60%	UAAUUUCUACUAAGUGUAGAUCCACCAGCACUUCAGCGUUC
WT_N_60%	GAACGCTGAAGTGCTGGTGGCAAA
NO.1(3'18)_60%	TGAAGTGCTGGTGGCAAA
NO.2 (3'17)_60%	GAAGTGCTGGTGGCAAA
NO.3 (3'16)_60%	AAGTGCTGGTGGCAAA
NO.4 (3'15)_60%	AGTGCTGGTGGCAAA
NO.5 (3'14)_60%	GTGCTGGTGGCAAA
NO.6 (3'13)_60%	TGCTGGTGGCAAA
NO.7 (3'12)_60%	GCTGGTGGCAAA
NO.8 (3'11)_60%	CTGGTGGCAAA
NO.9 (3'10)_60%	TGGTGGCAAA
NO.9 (5'14)_60%	GAACGCTGAAGTGC
NO.8 (5'13)_60%	GAACGCTGAAGTG
NO.7 (5'12)_60%	GAACGCTGAAGT
NO.6 (5'11)_60%	GAACGCTGAAG
NO.5 (5'10)_60%	GAACGCTGAA
NO.4 (5'9)_60%	GAACGCTGA
NO.3 (5'8)_60%	GAACGCTG
NO.2 (5'7)_60%	GAACGCT
NO.1 (5'6)_60%	GAACGC

Supplementary Table S7. Sequences in N-system (40% GC content) including crRNA and ssDNA activators.

Name	Sequence (5'-3')
crRNA_N_40%	UAAUUUCUACUAAGUGUAGAUACAACAGAACUUCAGAGUUC
WT_N_40%	GAACTCTGAAGTTCTGTTGTCAAA
NO.1 (3'18)_40%	TGAAGTTCTGTTGTCAAA
NO.2 (3'17)_40%	GAAGTTCTGTTGTCAAA
NO.3 (3'16)_40%	AAGTTCTGTTGTCAAA
NO.4 (3'15)_40%	AGTTCTGTTGTCAAA
NO.5 (3'14)_40%	GTTCTGTTGTCAAA
NO.6 (3'13)_40%	TTCTGTTGTCAAA
NO.7 (3'12)_40%	TCTGTTGTCAAA
NO.8 (3'11)_40%	CTGTTGTCAAA
NO.9 (3'10)_40%	TGTTGTCAAA
NO.9 (5'14)_40%	GAACTCTGAAGTTC
NO.8 (5'13)_40%	GAACTCTGAAGTT
NO.7 (5'12)_40%	GAACTCTGAAGT
NO.6 (5'11)_40%	GAACTCTGAAG
NO.5 (5'10)_40%	GAACTCTGAA
NO.4 (5'9)_40%	GAACTCTGA
NO.3 (5'8)_40%	GAACTCTG
NO.2 (5'7)_40%	GAACTCT
NO.1 (5'6)_40%	GAACTC

Supplementary Table S8: Sequences in E-system (70% GC content) including crRNA and ssDNA activators.

Name	Sequence (5'-3')
crRNA_E_70%	UAAUUUCUACUAAGUGUAGAUGUGGUACCCCUGCCAGCCAC
WT_E_70%	GTGGCTGGCAGGGGTACCACGAAA
NO.1 (3'18)_70% to NO.9 (3'10)_70%	Reference NO.1(3'18)_60% to NO.9(3'10)_60%
NO.9 (5'16)_70%	GTG GCT GGC AGG GGT A
NO.8 (5'15)_70%	GTG GCT GGC AGG GGT
NO.7 (5'14)_70%	GTG GCT GGC AGG GG
NO.6 (5'13)_70%	GTG GCT GGC AGG G
NO.5 (5'12)_70%	GTG GCT GGC AGG
NO.4 (5'11)_70%	GTG GCT GGC AG
NO.3 (5'10)_70%	GTG GCT GGC A
NO.2 (5'9)_70%	GTG GCT GGC
NO.1 (5'8)_60%	GTAACTGG

Supplementary Table S9. Sequences in E-system (60% GC content) including crRNA and ssDNA activators.

Name	Sequence (5'-3')
crRNA_E_60%	UAAUUUCUACUAAGUGUAGAUGUGGUACCCCUGCCAGUUAC
WT_E_60%	GTAACTGGCAGGGGTACCACGAAA
NO.1 (3'18)_60%	GGCAGGGGTACCACGAAA
NO.2 (3'17)_60%	GCAGGGGTACCACGAAA
NO.3 (3'16)_60%	CAGGGGTACCACGAAA
NO.4 (3'15)_60%	AGGGGTACCACGAAA
NO.5 (3'14)_60%	GGGGTACCACGAAA
NO.6 (3'13)_60%	GGGTACCACGAAA
NO.7 (3'12)_60%	GGTACCACGAAA
NO.8 (3'11)_60%	GTACCACGAAA
NO.9 (3'10)_60%	TACCACGAAA
NO.9 (5'16)_60%	GTAACTGGCAGGGGTA
NO.8 (5'15)_60%	GTAACTGGCAGGGGT
NO.7 (5'14)_60%	GTAACTGGCAGGGG
NO.6 (5'13)_60%	GTAACTGGCAGGG
NO.5 (5'12)_60%	GTAACTGGCAGG
NO.4 (5'11)_60%	GTAACTGGCAG
NO.3 (5'10)_60%	GTAACTGGCA
NO.2 (5'9)_60%	GTAACTGGC
NO.1 (5'8)_60%	GTAACTGG

Supplementary Table S10. Sequences in E-system (40% GC content) including crRNA and ssDNA activators.

Name	Sequence (5'-3')
crRNA_E_40%	UAAUUUCUACUAAGUGUAGAUGUGGUAUUCUUGCUAGUUAC
WT_E_40%	GTAACTAGCAAGAATACCACGAAA
NO.1 (3'18)_40%	AGCAAGAATACCACGAAA
NO.2 (3'17)_40%	GCAAGAATACCACGAAA
NO.3 (3'16)_40%	CAAGAATACCACGAAA
NO.4 (3'15)_40%	AAGAATACCACGAAA
NO.5 (3'14)_40%	AGAATACCACGAAA
NO.6 (3'13)_40%	GAATACCACGAAA
NO.7 (3'12)_40%	AATACCACGAAA
NO.8 (3'11)_40%	ATACCACGAAA
NO.9 (3'10)_40%	TACCACGAAA
NO.9 (5'16)_40%	GTAACTAGCAAGAATA
NO.8 (5'15)_40%	GTAACTAGCAAGAAT
NO.7 (5'14)_40%	GTAACTAGCAAGAA
NO.6 (5'13)_40%	GTAACTAGCAAGA
NO.5 (5'12)_40%	GTAACTAGCAAG
NO.4 (5'11)_40%	GTAACTAGCAA
NO.3 (5'10)_40%	GTAACTAGCA
NO.2 (5'9)_40%	GTAACTAGC
NO.1 (5'8)_40%	GTAACTAG

Supplementary Table S11. Sequences of crRNA, 3′(x) segments and 5′(y) segments in extended N-system.

Name	Sequence (5'-3')
crRNA_N extensioin	UAAUUUCUACUAAGUGUAGAUCCCCCAGCGCUUCAGCGUUCuuauu
WT_N extension	aataaGAACGCTGAAGCGCTGGGGGCAAA
NO.1 (3'18) to NO.9 (3'10)	Reference NO.1 (3'18)_70% to NO.9 (3'10)_70%
NO.9 (5'19)	aataaGAACGCTGAAGCGC
NO.8 (5'18)	aataaGAACGCTGAAGCG
NO.7 (5'17)	aataaGAACGCTGAAGC
NO.6 (5'16)	aataaGAACGCTGAAG
NO.5 (5'15)	aataaGAACGCTGAA
NO.4 (5'14)	aataaGAACGCTGA
NO.3 (5'13)	aataaGAACGCTG
NO.2 (5'12)	aataaGAACGCT
NO.1 (5'11)	aataaGAACGC

Supplementary Table S12. Sequences of crRNA, 3′(x) segments and 5′(y) segments in shortened P-system.

Name	Sequence (5'-3')
crRNA_P shorting	AAUUUCUACUCUUGUAGAUGCCAGGGACGAAGCGCAAGU
WT_P_shorting	ACTTGCGCTTCGTCCCTGGCGAAA
NO.1 (3'18)	GCTTCGTCCCTGGCGAAA
NO.2 (3'17)	CTTCGTCCCTGGCGAAA
NO.3 (3'16)	TTCGTCCCTGGCGAAA
NO.4 (3'15)	TCGTCCCTGGCGAAA
NO.5 (3'14)	CGTCCCTGGCGAAA
NO.6 (3'13)	GTCCCTGGCGAAA
NO.7 (3'12)	TCCCTGGCGAAA
NO.8 (3'11)	CCCTGGCGAAA
NO.9 (3'10)	CCTGGCGAAA
NO.9 (5'14)	ACTTGCGCTTCGTC
NO.8 (5'13)	ACTTGCGCTTCGT
NO.7 (5'12)	ACTTGCGCTTCG
NO.6 (5'11)	ACTTGCGCTTC
NO.5 (5'10)	ACTTGCGCTT
NO.4 (5'9)	ACTTGCGCT
NO.3 (5'8)	ACTTGCGC
NO.2 (5'7)	ACTTGCG
NO.1 (5'6)	ACTTGC

Supplementary Table S13. The hairpin sequences used in the cleaved experiments.

Name	Sequence# (5'-3')	Length
**H0**	GAAGCGCTGGGGGCAAA*TTTT*TTGCCCCCAGCGCTTC	38
H0-1	GAAGCGCTGGGGGCAAA*ATTTTTTTTTTA*TTTGCCCCCAGC GCTTC	46
H0-2	GGGCAAA*TTTT*TTTGCCC	18
H0-3	GCAAA*ATTTTTTTTTTA*TTTGC	22
H0-4	GAAGCGCTGGGGGCAAA*TTTT*TTGCCCCCAGCGCTTCTTA TTAT	45
H0-5	GCTGGGGGCAAA*TTTT*TTGCCCCCAGCTTATTAT	35
Probe DNA	TTATTATT	8

#DNAs have both 5'FAM and 3'BHQ modifications. The red color indicates adding 3' overhang sequences. The italic underline marks the hairpin structure region.

Supplementary Table S14. Sequences used in the detection of tumor cell lines.

Name	Sequence (5'-3')
crRNA1	UAAUUUCUACUAAGUGUAGAUCCCCCAGCGCUUCAGCGUUC
crRNA2	UAAUUUCUACUCUUGUAGAUGAUUUUCUUCCUUUUGUUCA
BRCA-1	GAACAAAAGGAAGAAAATCA
BRCA-2	GAACAAAATCCTCCAAACTC
miRNA-21	TAG CTT ATC AGA CTG ATG TTG A
miRNA-let7a	TGA GGT AGT AGG TTG TAT AGT T
miRNA-141	TAACACTGTCTGGTAAAGATGG
miRNA-let7d	AGAGGTAGTAGGTTGCATAGTT
miRNA-7e	TGAGGTAGGAGGTTGTATAGTT
miRNA-122	TGGAGTGTGACAATGGTGTTTG

Supplementary Table S15. Comparison of Cas12a-based nucleic acid detection methods.

Detecting method	Object	Strategy	Readout*	Detecting time (min)	LOD
Electrochemical platform[1]	HPV-16	Label on carbon electrode	HRP	120	100 fM
Electrochemical platform[2]	HPV-16	Label on metal electrode	MB	30	50 pM
Electrochemical platform[3]	PB-19	Label on metal electrode	MB	-	10 fM
CONAN[4]	hepatitis B	One-pot	Fluo	180 (5 aM)<br>30 (1 fM)	5 aM
Metal-enhanced Fuorescence[5]	*BRCA*-1	Au-nanoparticles	Fluo/ Colorimetry	30	1 fM
CALSA	Synthetic plasmid	One-pot	Fluo	50	25 fM
	BRCA-1			55	4.7 fM

*HRP means Horseradish Peroxidase, MB means methylene blue, Fluo means Fluorescence.

References

1. Su,J., Ke,Y., Maboyi,N., Zhi,X., Yan,S., Li,F., Zhao,B., Jia,X., Song,S. and Ding,X. (2021) CRISPR/Cas12a Powered DNA Framework-Supported Electrochemical Biosensing Platform for Ultrasensitive Nucleic Acid Analysis. Small Methods, **5**, 2100935.
2. Dai,Y., Somoza,R.A., Wang,L., Welter,J.F., Li,Y., Caplan,A.I. and Liu,C.C. (2019) Exploring the *Trans*-Cleavage Activity of CRISPR-Cas12a (cpf1) for the Development of a Universal Electrochemical Biosensor. Angew. Chemie Int. Ed., **58**, 17399–17405.
3. Xu,W., Jin,T., Dai,Y. and Liu,C.C. (2020) Surpassing the detection limit and accuracy of the electrochemical DNA sensor through the application of CRISPR Cas systems. Biosens. Bioelectron., **155**, 112100.
4. Shi,K., Xie,S., Tian,R., Wang,S., Lu,Q., Gao,D., Lei,C., Zhu,H. and Nie,Z. (2021) A CRISPR-Cas autocatalysis-driven feedback amplification network for supersensitive DNA diagnostics. Sci. Adv., **7**, eabc7802.
5. Choi,J.H., Lim,J., Shin,M., Paek,S.H. and Choi,J.W. (2021) CRISPR-Cas12a-Based Nucleic Acid Amplification-Free DNA Biosensor via Au Nanoparticle-Assisted Metal-Enhanced Fluorescence and Colorimetric Analysis. Nano Lett., **21**, 693–699.

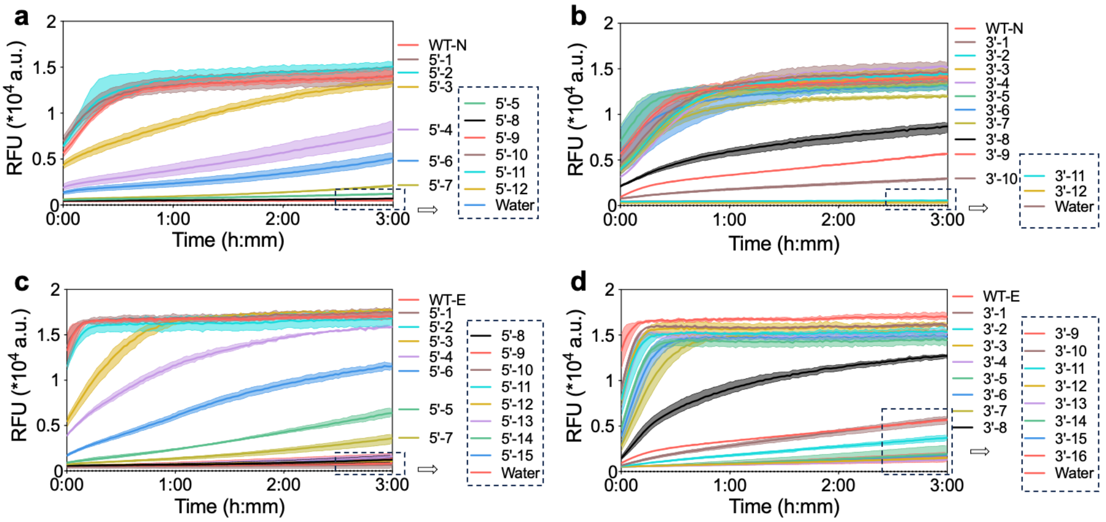

Supplementary Figure S1. Time-dependent fluorescence signals of truncated ssDNA from both 5’ and 3’ ends of N-TS (a, b) and E-TS (c, d) in activating LbCas12a *trans*-cleavage activity. The WT represented the fluorescence signal of full-length activator.

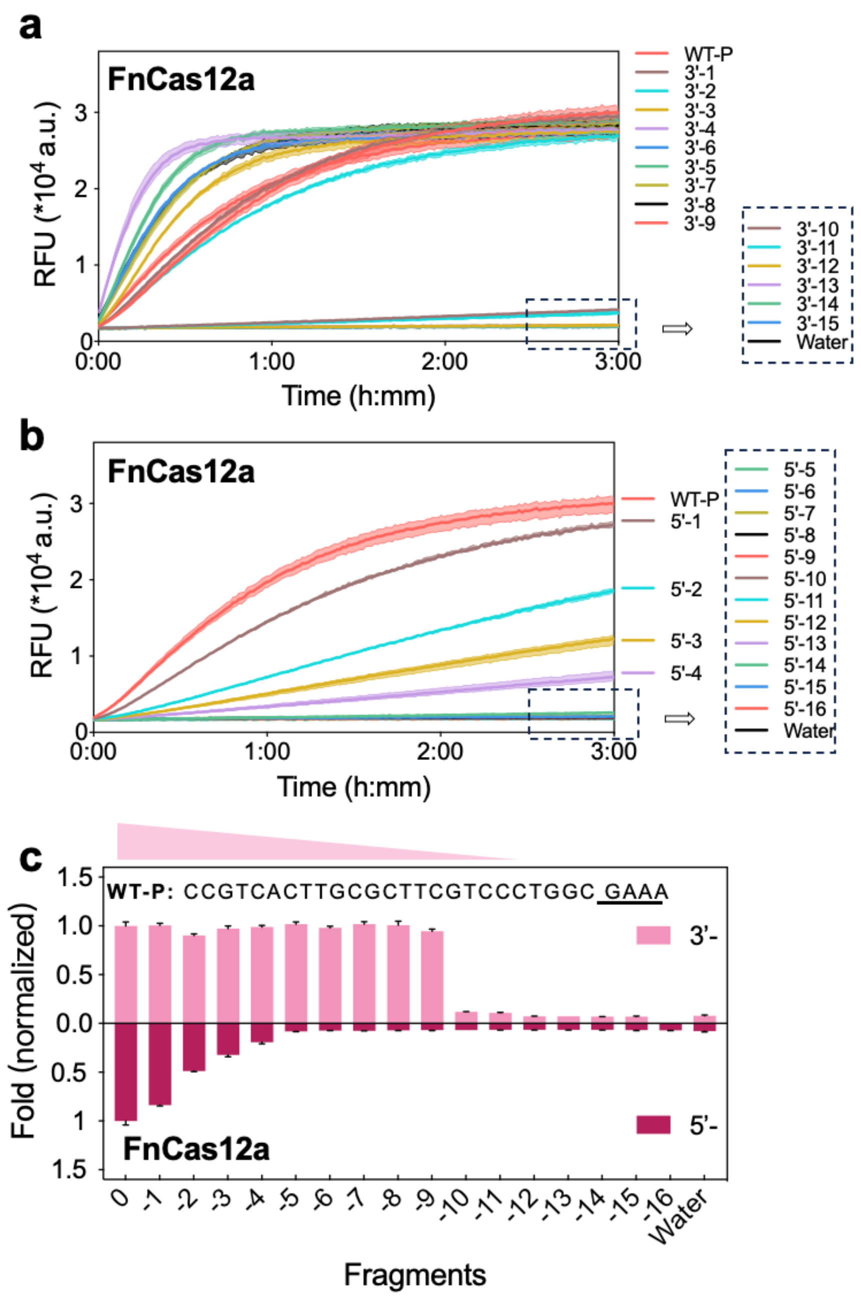

Supplementary Figure S2. Tolerance of FnCas12a to the truncated ssDNA from both 5' and 3' ends of P-TS for *trans*-cleavage activity. (a, b) Time-dependent fluorescence signals of truncated ssDNA from both 3' (a) and 5' ends (b) of P-TS in activating FnCas12a *trans*-cleavage activity. The WT represented the fluorescence signal of full-length activator. (c) Bar graph showing the relative fluorescence fold change of truncated ssDNA from both 3' and 5' ends of P-TS in activating FnCas12a *trans*-cleavage activity. The fold in fluorescence was normalized by taking the ratio of positive-group fluorescence signal of a sample with the full-length activator (WT-P) to the sample with truncated activators. Data are expressed as mean ± SD (n = 3).

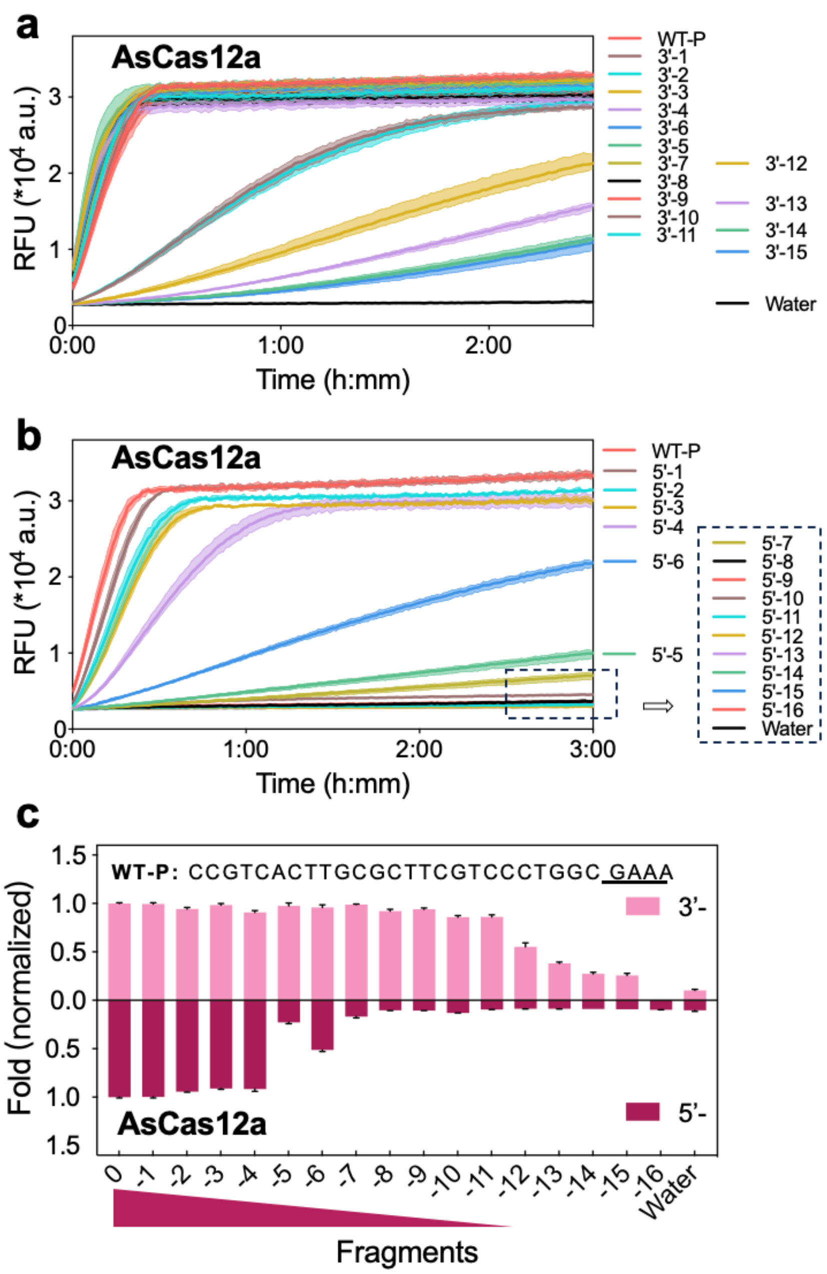

Supplementary Figure S3. Tolerance of AsCas12a to the truncated ssDNA from both 5' and 3' ends of P-TS for *trans*-cleavage activity. (a,b) Time-dependent fluorescence signals of truncated ssDNA from both 3' (a) and 5' ends (b) of P-TS in activating AsCas12a *trans*-cleavage activity. The WT represented the fluorescence signal of full-length activator. (c) Bar graph showing the relative fluorescence fold change of truncated ssDNA from both 3' and 5' ends of P-TS in activating AsCas12a *trans*-cleavage activity. The fold in fluorescence was normalized by taking the ratio of positive-group fluorescence signal of a sample with the full-length activator (WT-P) to the sample with truncated activators. Data are expressed as mean ± SD (n = 3).

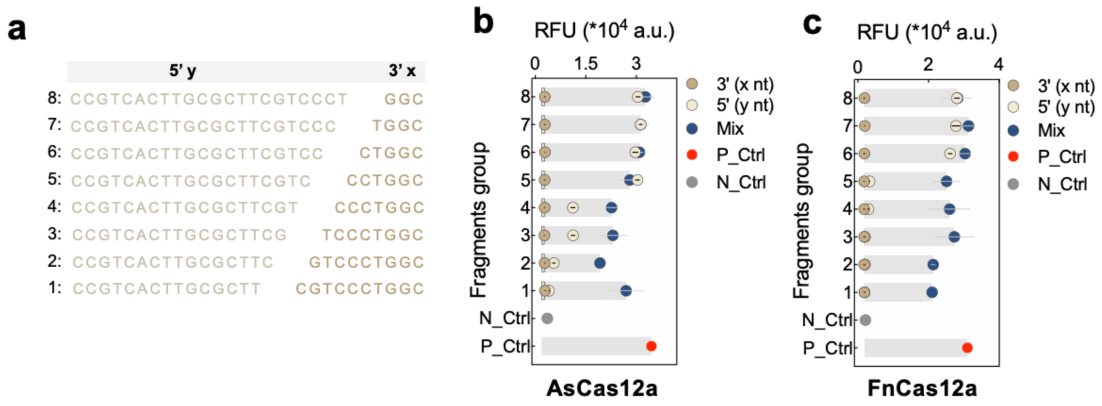

Supplementary Figure S4. Switching AsCas12a and FnCas12a *trans*-cleavage activity via combinations of two truncated ssDNA segments. (a) The sequences of truncated ssDNA segments 5'(y) and 3'(x) from P-TS. (b, c) Truncated ssDNA segments activation effects on AsCas12a and FnCas12a, both individually and in combination. Full-length ssDNA served as the positive control (P_Ctrl, 100% activation effect), while RNase-free water served as the negative control (N_Ctrl) to activate Cas12a. Data are expressed as mean ± SD (n = 3).

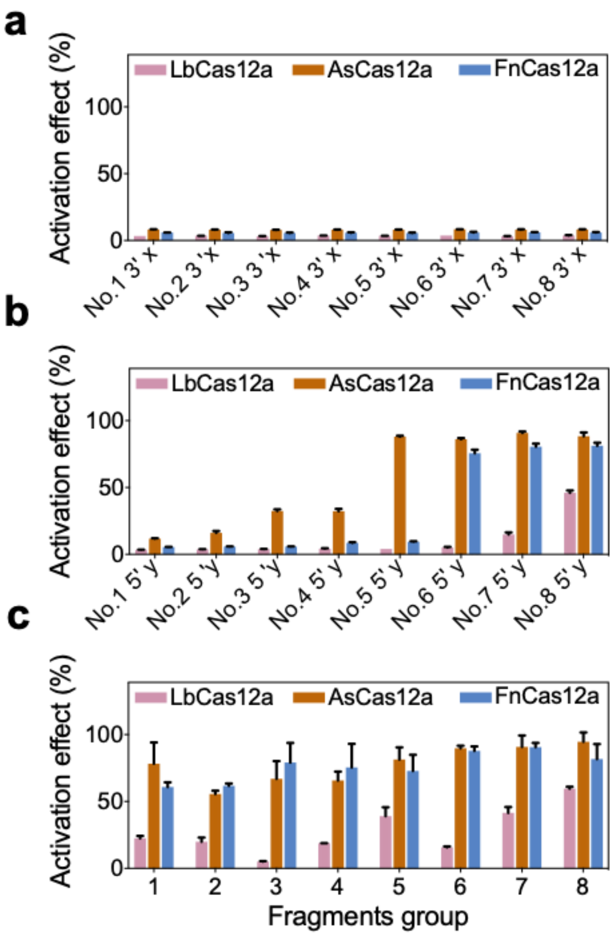

Supplementary Figure S5. Comparing the activation effect of the individual 3'x (a) or 5'y (b) segments and their combinations (c) on LbCas12a, AsCas12a, and FnCas12a respectively. Data are expressed as mean ± SD (n = 3).

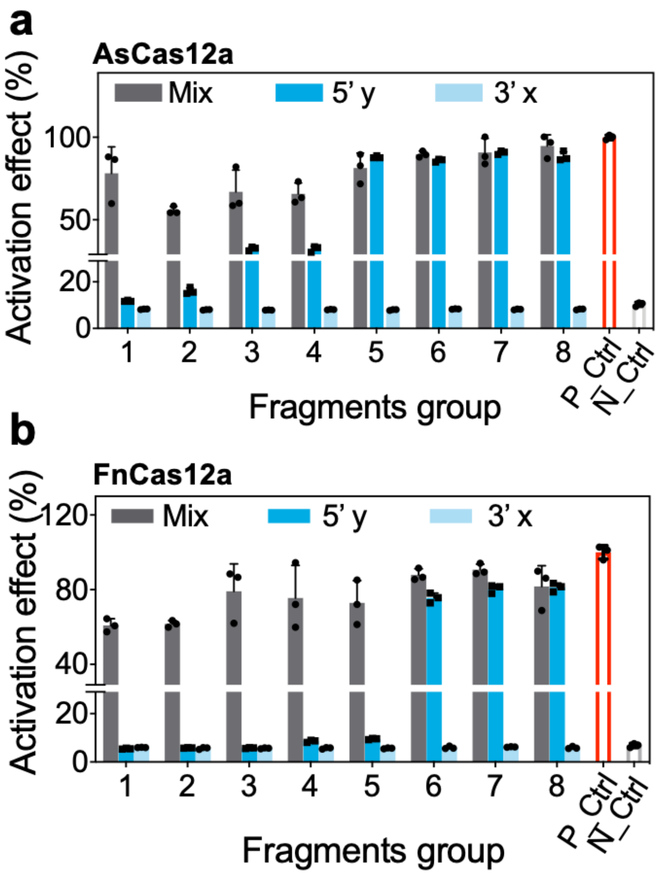

Supplementary Figure S6. The activation effect of the truncated ssDNA alone as well as the combination of the "x" and "y" segments from P-TS on AsCas12a (a) and FnCas12a (b), respectively. Truncated ssDNA segments activation effects on AsCas12a and FnCas12a, both individually and in combination. Full-length ssDNA served as the positive control (P_Ctrl, 100% activation effect), while RNase-free water served as the negative control (N_Ctrl) to activate Cas12a. Data are expressed as mean ± SD (n = 3).

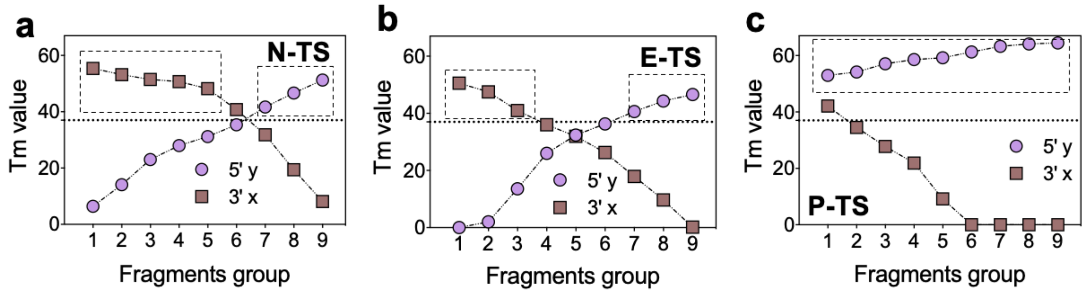

Supplementary Figure S7: The melting temperatures (Tm) of the ssDNA combinations in the "N"(a), "E" (b) and "P" (c) systems. The dashed frames highlight the "x" or "y" segments with Tm values above 37 °C.

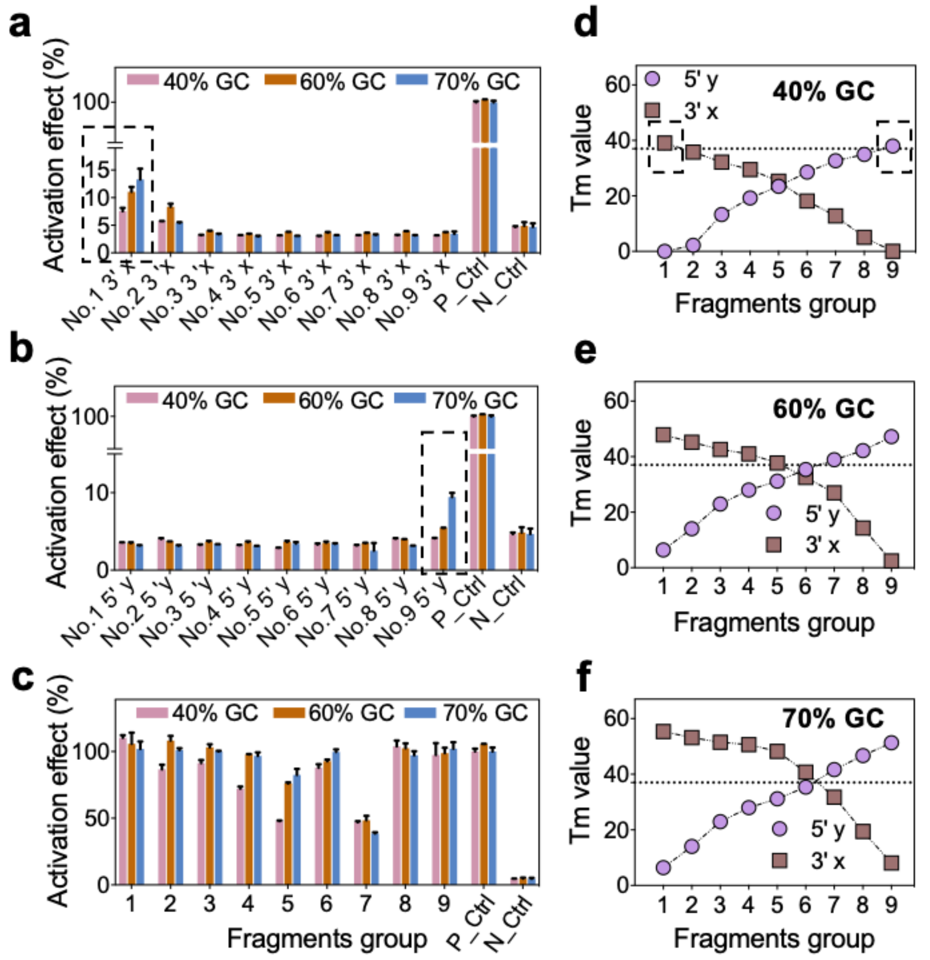

Supplementary Figure S8. The activation effect and Tm values of different segments alone from N-TS with 40%, 60%, and 70% GC content as well as the segment combinations on LbCas12a (a, b, and c). Full-length ssDNA with different GC content served as the positive control (P_Ctrl, 100% activation effect), while RNase-free water served as the negative control (N_Ctrl) to activate Cas12a. The Tm value of different segments from N-TS with 40% (d), 60% (e) and 70% GC (f) content. Data are expressed as mean ± SD (n = 3).

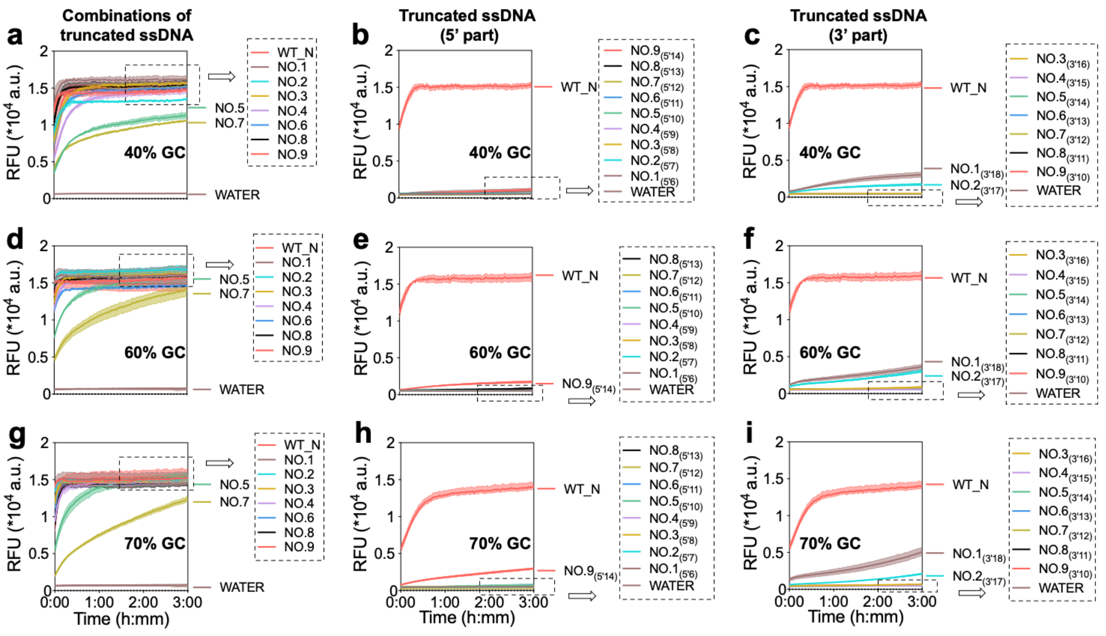

Supplementary Figure S9. Time-dependent fluorescence signals of the truncated ssDNA alone with 40%, 60% and 70% GC content as well as the combination of the "x" and "y" segments from N-TS on LbCas12a.

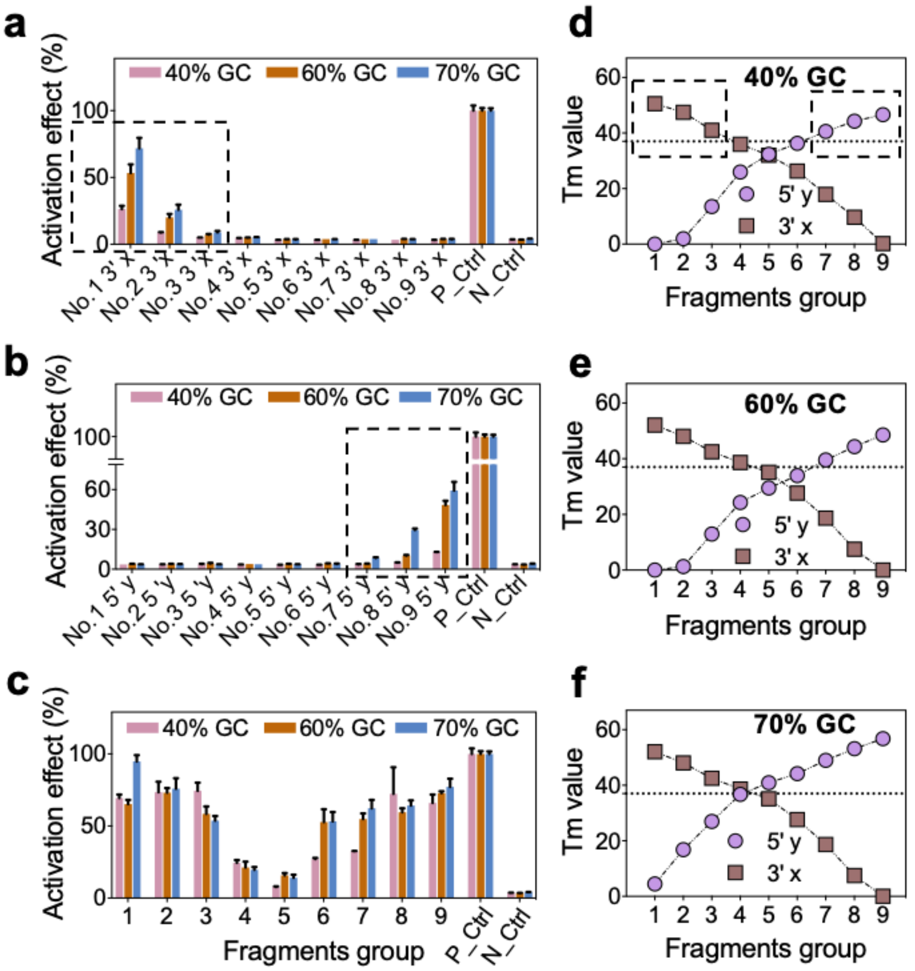

Supplementary Figure S10. The activation effect and Tm values of different segments alone from E-TS with 40%, 60%, and 70% GC content as well as the segment combinations on LbCas12a (a, b, and c). Full-length ssDNA with different GC content served as the positive control (P_Ctrl, 100% activation effect), while RNase-free water served as the negative control (N_Ctrl) to activate Cas12a. The Tm value of different segments from E-TS with 40% (d), 60% (e) and 70% GC (f) content. Data are expressed as mean ± SD (n = 3).

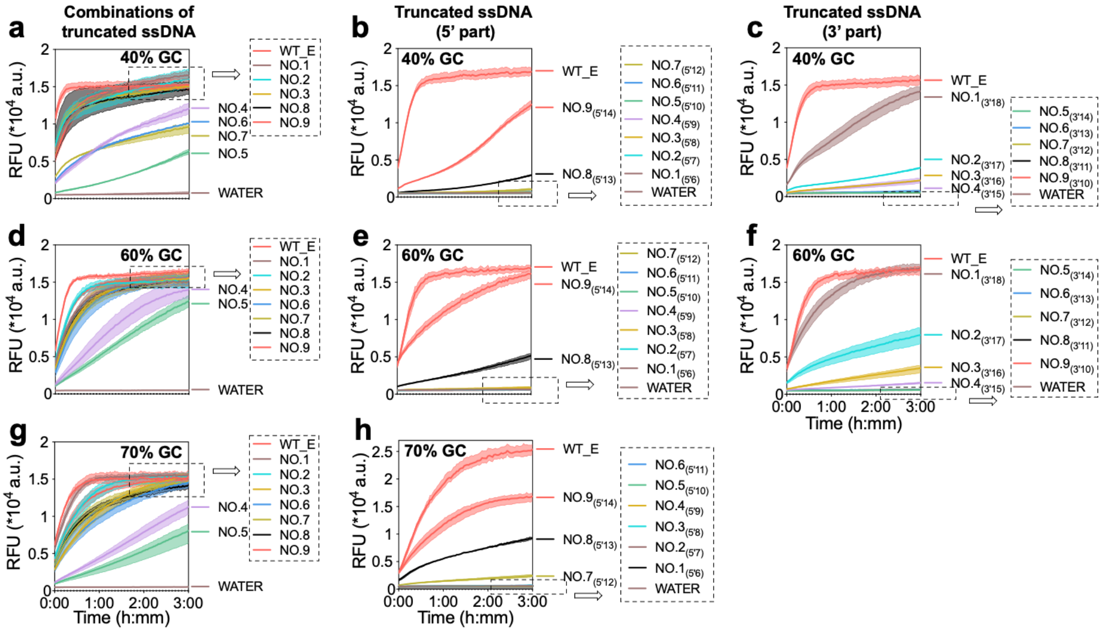

Supplementary Figure S11. Time-dependent fluorescence signals of the truncated ssDNA alone with 40%, 60% and 70% GC content as well as the combination of the "x" and "y" segments from E-TS on LbCas12a. The WT represented the fluorescence signal of full-length activator.

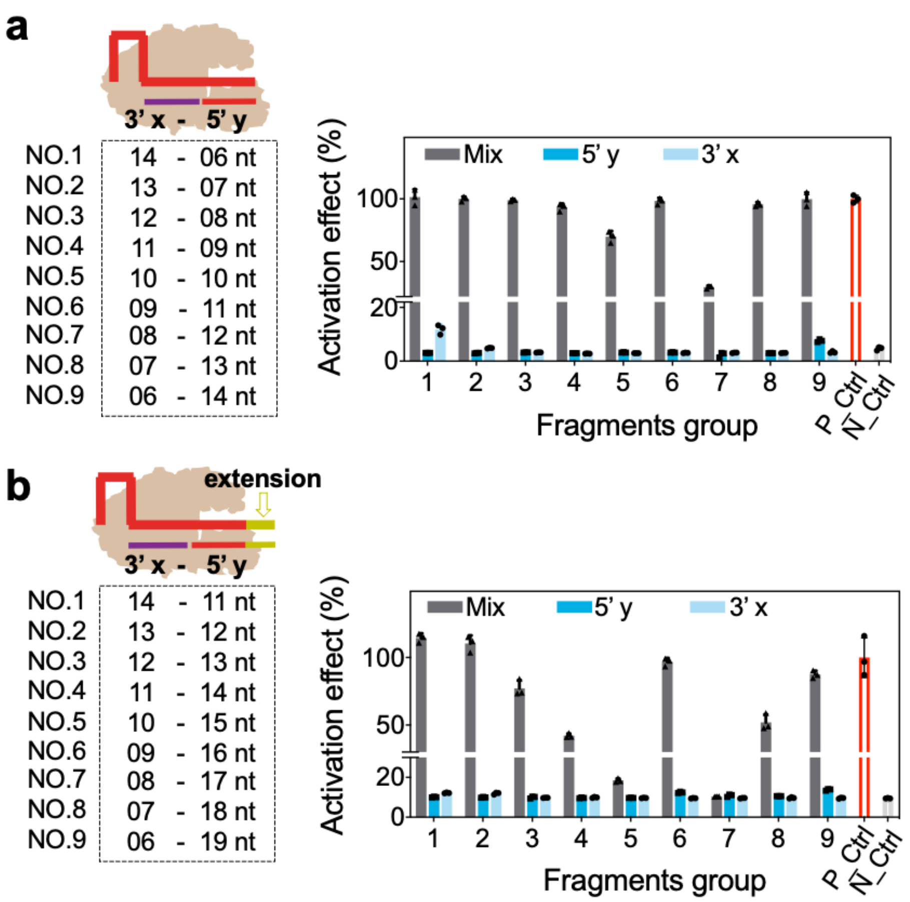

Supplementary Figure S12. Comparing the activation effect for LbCas12a of the 3' (x) and 5' (y) segments, as well as their combinations from "N" system (a) and extended "N" system (b) respectively. Full-length ssDNA served as the positive control (P_Ctrl, 100% activation effect), while RNase-free water served as the negative control (N_Ctrl) to activate Cas12a. Data are expressed as mean ± SD (n = 3).

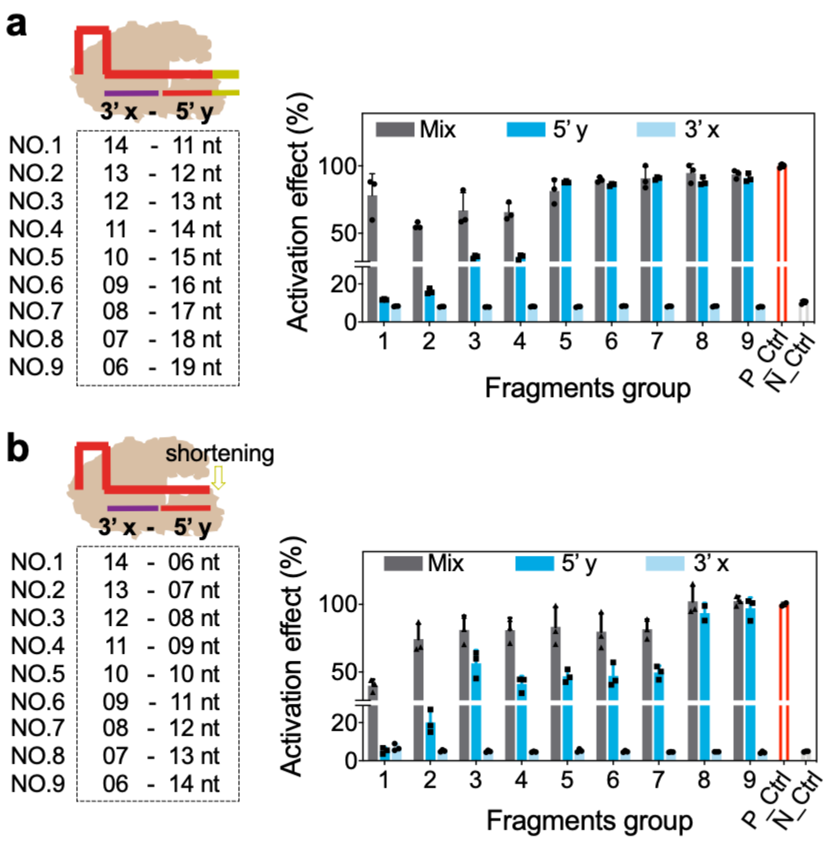

Supplementary Figure S13. Comparing the activation effect for AsCas12a of the 3' (x) and 5' (y) segments, as well as their combinations from "P" system (a) and shortened "P" system (b) respectively. Full-length ssDNA served as the positive control (P_Ctrl, 100% activation effect), while RNase-free water served as the negative control (N_Ctrl) to activate Cas12a. Data are expressed as mean ± SD (n = 3).

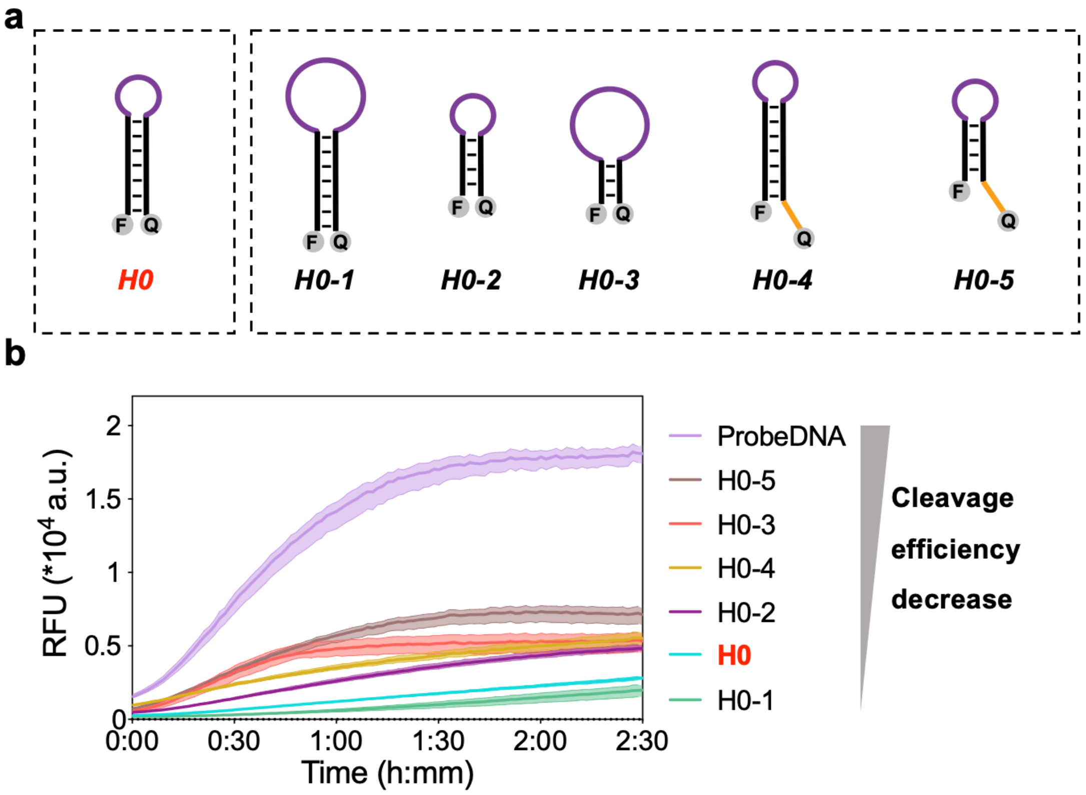

Supplementary Figure S14. The cleavage efficiency of different hairpin DNA by activated LbCas12a. (a) The structures of hairpin DNAs. (b) Time-response fluorescence signal of activated LbCas12a cleaving Probe DNA and hairpin DNAs.

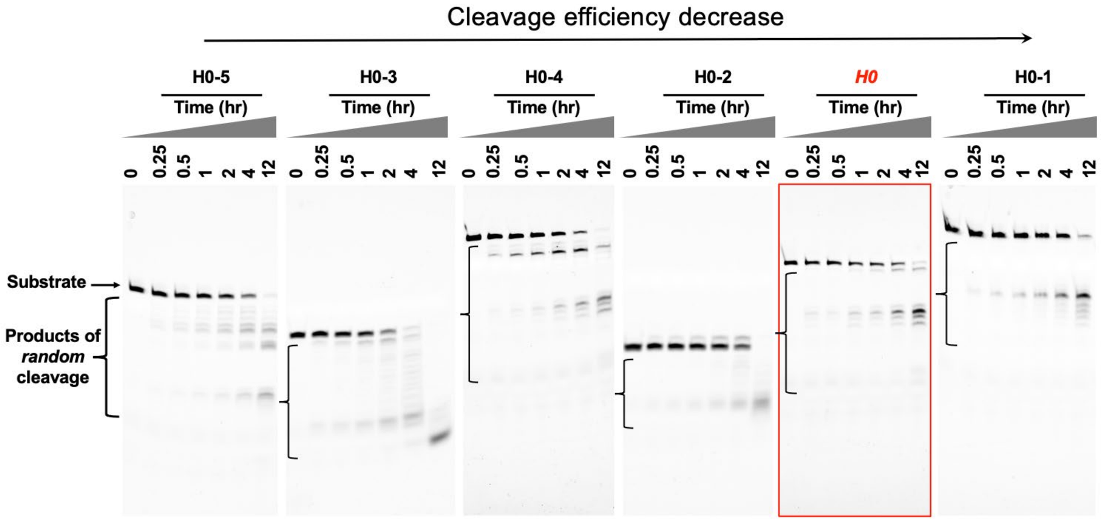

Supplementary Figure S15. High-resolution PAGE analysis of *trans*-cleavage of hairpin DNAs by LbCas12a. Red box highlighted the original hairpin DNA H0 in this study.

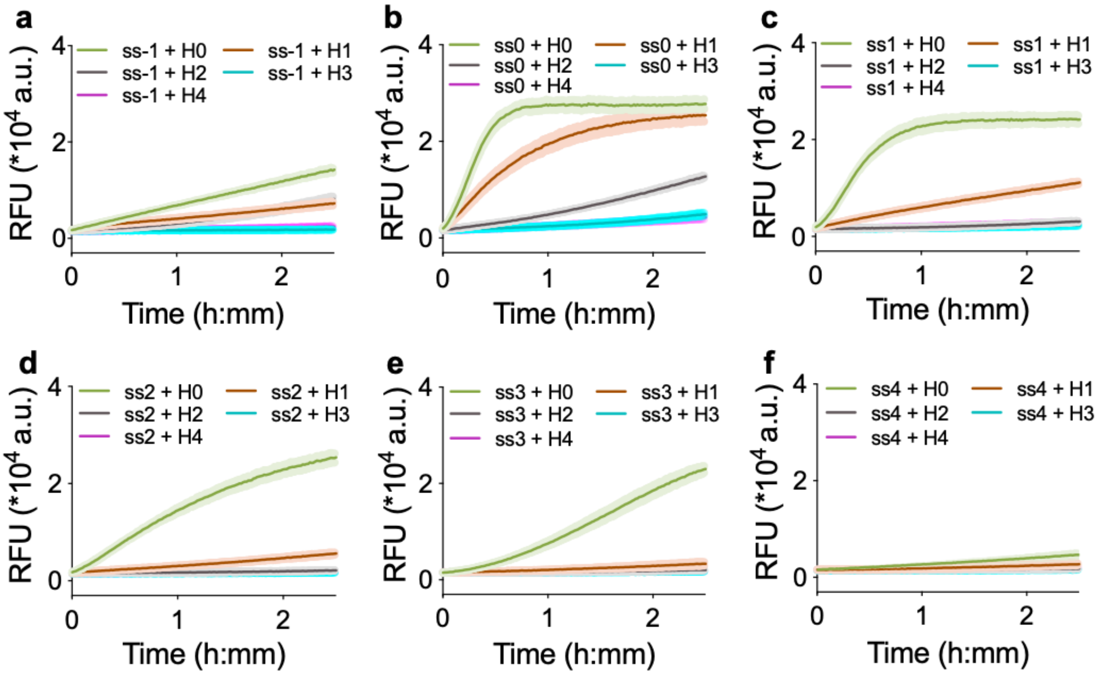

Supplementary Figure S16. Switching LbCas12a activity through structural variations of two activator segments. (a-f) Time-dependent fluorescence signals of LbCas12a *trans*-cleavage activity for different combinations of ss-1 (a) with H0 to H4, ss0 (b) with H0 to H4, ss1 (c) with H0 to H4, ss2 (d) with H0 to H4, ss3 (e) with H0 to H4, and ss4 (f) with H0 to H4.

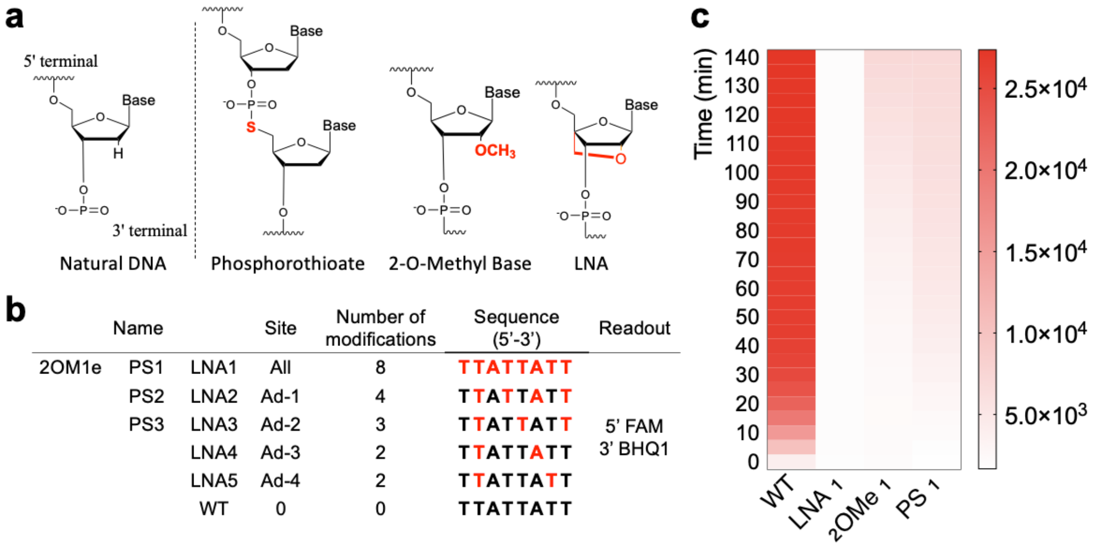

Supplementary Figure S17. The *trans*-cleavage activity of LbCas12a to the modified reporter via fluorescence measurement. (a) Chemical structure of natural, phosphorothioate, 2'-OMeN and LNA modified DNA. (b) Different types of reporter DNA used in the fluorescence measurement. (c) Heat map showing the *trans*-cleavage activity of the Cas12a to full modification probes.

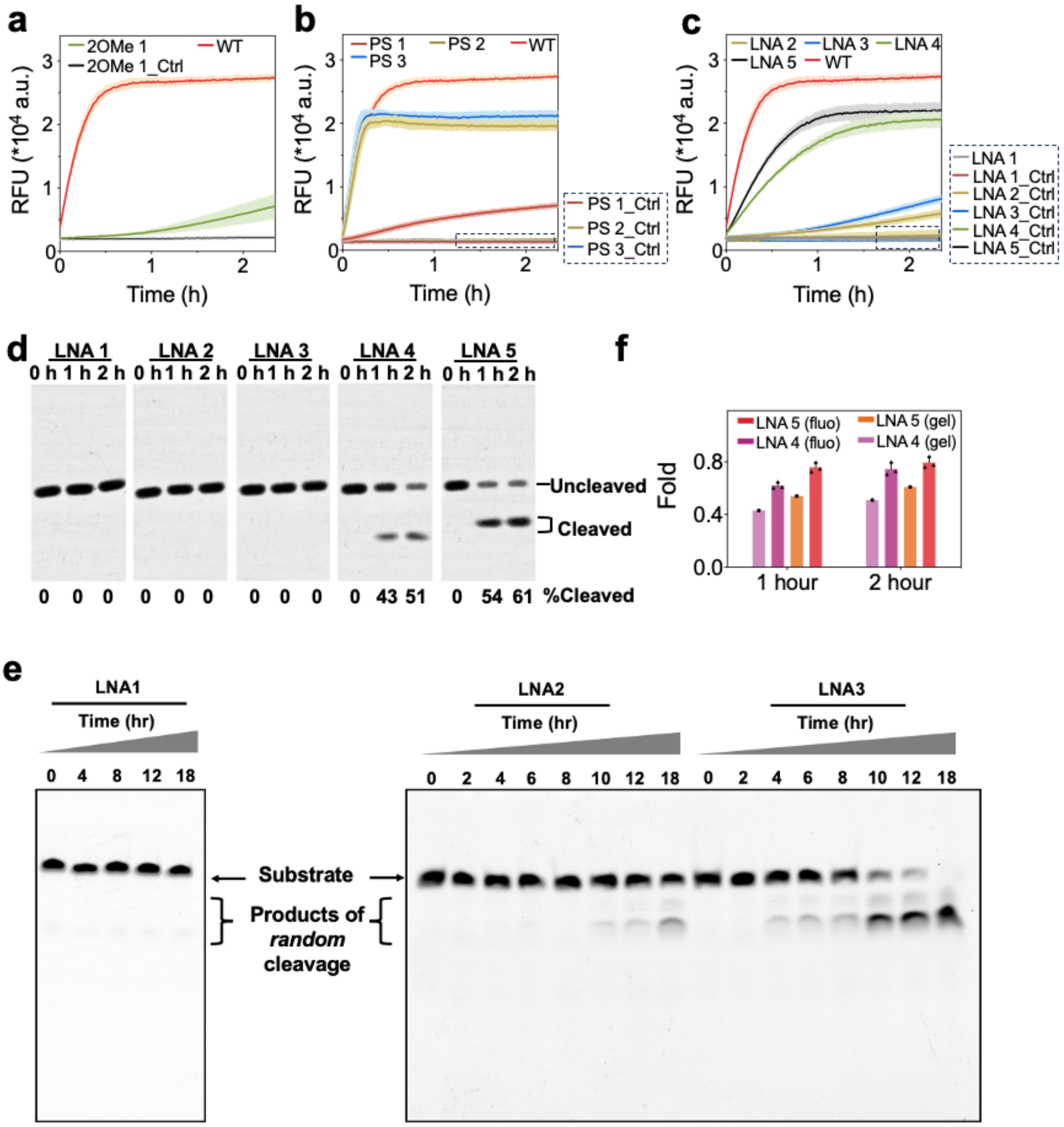

Supplementary Figure S18. The *trans*-cleavage activity of LbCas12a to the modified reporter via fluorescence measurement and high-resolution PAGE analysis. (a-c) Time-dependent fluorescence signals of LbCas12a *trans*-cleavage activity for 2'-OMeN (a), PS (b), and LNA modified reporter DNA (c). The WT referred to reporter DNA without modification, and the control samples (Ctrl) referred to reactions without activators. (d) High-resolution PAGE analysis of LNA1 to LNA 5. (e) High-resolution PAGE analysis of LNA1, LNA2, and LNA3 over 18 hours reaction. (f) The comparison of resistance effect by LNA 4 and LNA 5 between fluorescence measurement and high-resolution PAGE analysis.

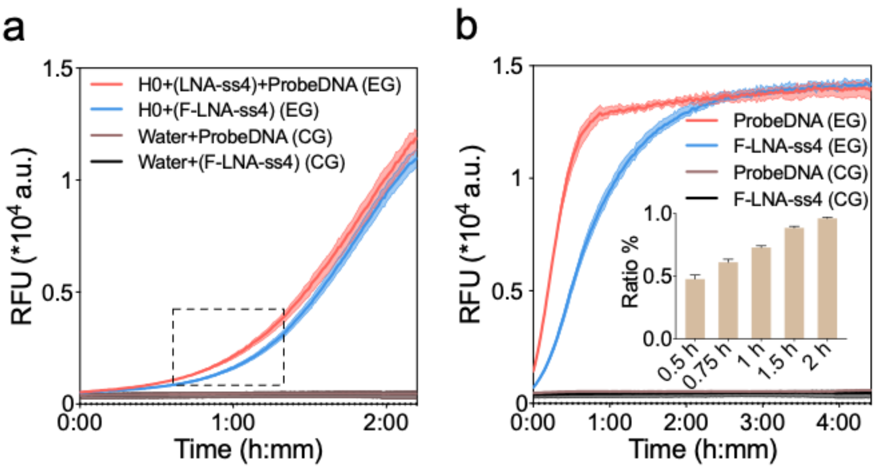

Supplementary Figure S19. Comparison of detection efficiency between fluorescently labeled LNA-ss 4 and classical fluorescently Probe DNA. (a) Comparison of detection efficiency using F-LNA-ss4 and Probe DNA in the CALSA method respectively. (b) Comparison of cleavage efficiency of F-LNA-ss4 and Probe DNA by the activated LbCas12a. Inset showing the cleavage efficiency ratio (F-LNA-ss4 vs. Probe DNA) at different timepoints. EG experimental group with H0 and LNA-ss4, and CG represented control group without H0 or LNA-ss4.

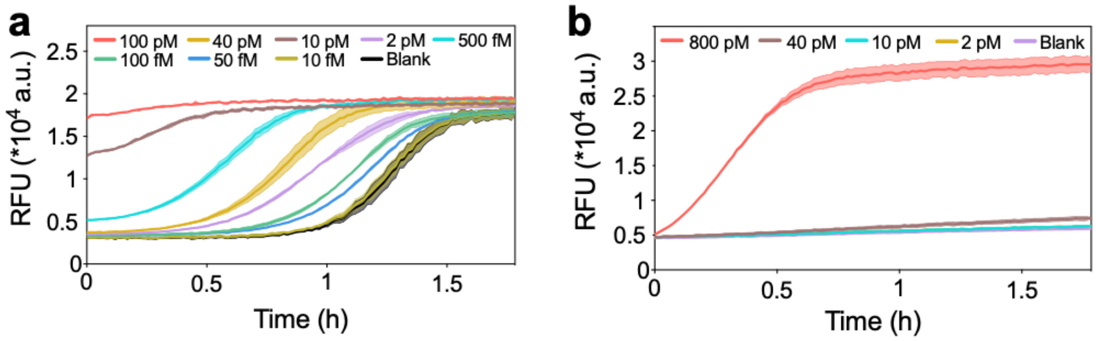

Supplementary Figure S20. Fluorescence responses of target plasmid at different concentrations in CALSA. (a) Time-dependent fluorescence signals in response to different concentrations ranging from 10 fM to 100 pM of target plasmid in CALSA. Blank represented samples without plasmid. (b) Time-dependent fluorescence signals in response to different concentrations ranging from 2 pM to 800 pM of target plasmid in the direct method with LbCas12a.

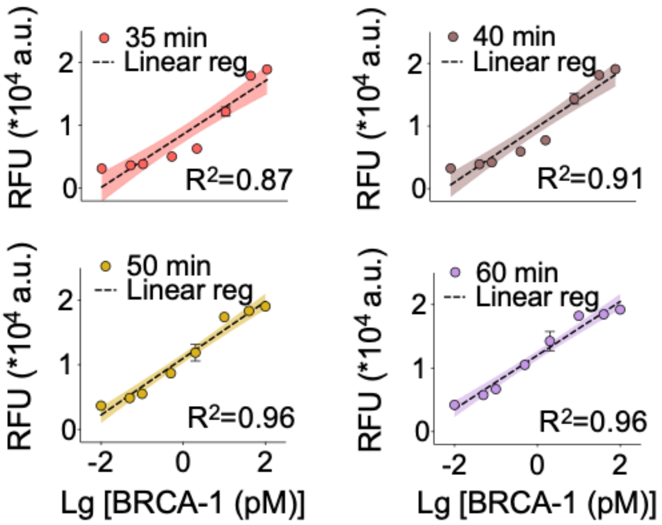

Supplementary Figure S21. Calibration curves depicting the fluorescence response of CALSA to target plasmid at different time intervals and concentrations.

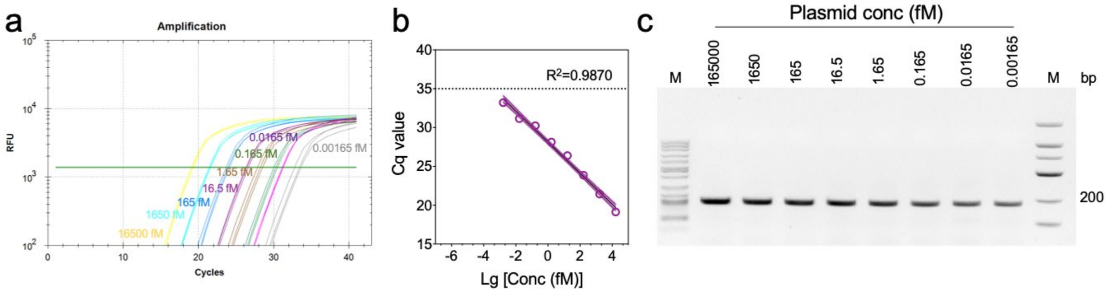

Supplementary Figure S22. Target plasmid detection by qPCR method. (a) Quantitative amplification curves of target plasmid (from 16.5 pM to 1.65 aM). (b) The calibration curve (Cq value *vs*. Concentration). (c) The agarose gel results of qPCR products.

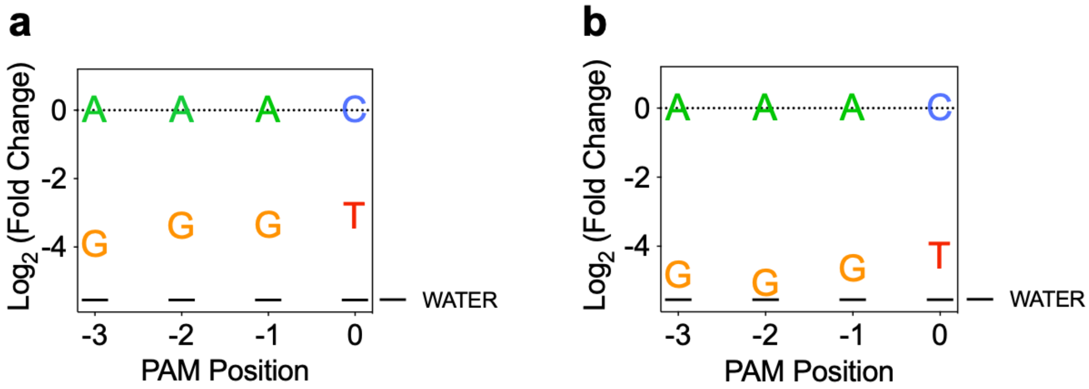

Supplementary Figure S23. Graphs illustrating the relative fold decrease in fluorescence of single (a) and double mutants (b) at the PAM region. Data are expressed as mean ± SD (n = 3).

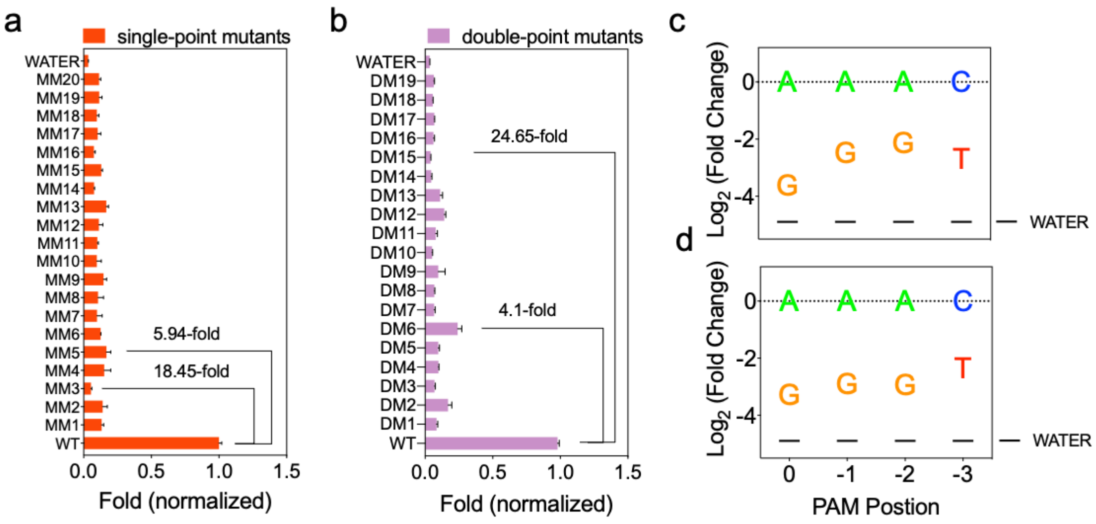

Supplementary Figure S24. The specificity of the conventional Cas12a approach. Graphs illustrating the relative fold decrease in fluorescence of single (a) and double mutants (b) at the PAM region. (a, b) Bar graphs showing the relative fluorescence fold change of the MM1-MM20 and DM1-DM19 mutant DNA activators compared to the WT activator in the conventional Cas12a detection method. (c, d) Graphs illustrating the relative fold decrease in fluorescence of single and double mutants at the PAM region in the conventional Cas12a detection method. The final concentration of mutant activators was 100 pM. Data are expressed as mean ± SD (n = 3).

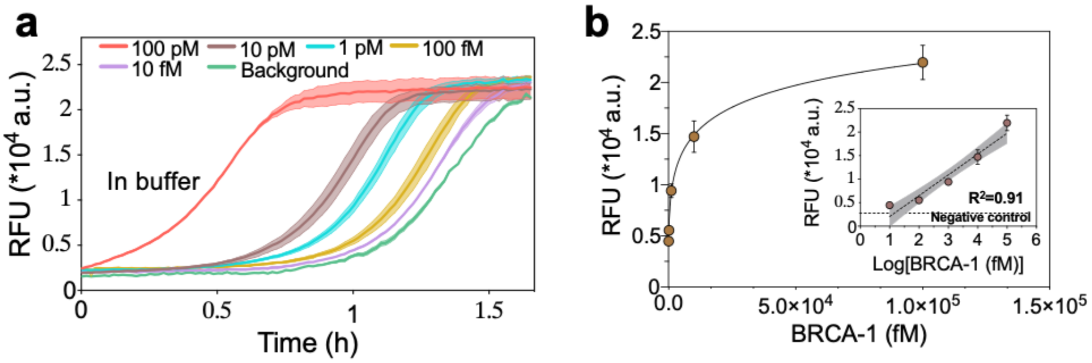

Supplementary Figure S25. Fluorescence responses of *BRCA-1* at different concentrations in 1xPBS with CALSA. (a) Time-dependent fluorescence signals in response to different concentration of *BRCA-1* ranging from 10 fM to 100 pM in CALSA. Background represented samples without *BRCA-1*. (b) Experimental analysis of time-dependent fluorescence signal changes in CALSA in response to different concentrations of *BRCA-1*, along with insets illustrating the corresponding calibration curve. Data are expressed as mean ± SD (n = 3).

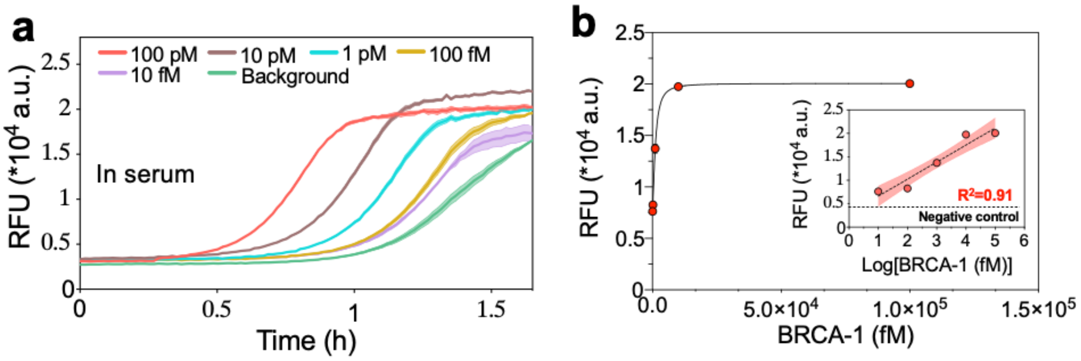

Supplementary Figure S26. Fluorescence responses of *BRCA-1* at different concentrations in 5% human serum with CALSA. (a) Time-dependent fluorescence signals in response to different concentration of *BRCA-1* ranging from 10 fM to 100 pM in CALSA. Background represented samples without *BRCA-1*. (b) Experimental analysis of time-dependent fluorescence signal changes in CALSA in response to different concentrations of *BRCA-1*, along with insets illustrating the corresponding calibration curve. Data are expressed as mean ± SD (n = 3).

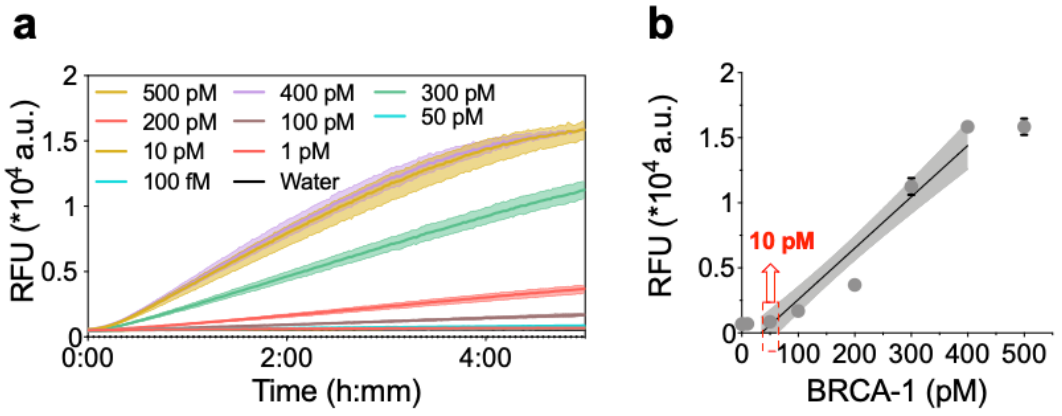

Supplementary Figure S27. Synthetic BRCA-1 detection by the direct Cas12a detection method. (a) Time-dependent fluorescence signals in response to different concentrations ranging from 100 fM to 500 pM of BRCA-1 in the direct method with LbCas12a. (b) Calibration curves illustrating the response of the direct Cas12a detection method to various concentrations of BRCA-1 (The gray shadow indicating the 95% confidence band and the red arrow highlighting the fitted minimal concentration of 10 pM).

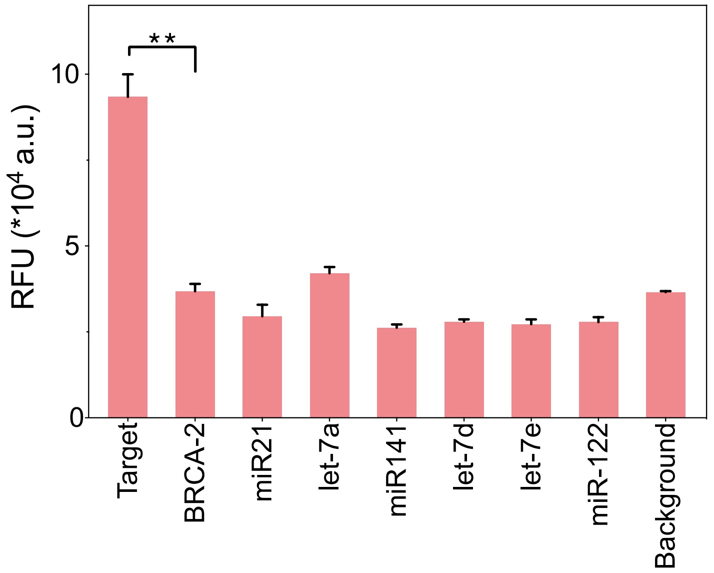

Supplementary Figure S28. Comparison of fluorescence intensities among different nucleic acids, including target BRCA-1 and interfering BRCA-2, miR-21, let-7a, miR-141, let-7d, let-7e as well as miR-122 for the verification of selective detection. Data are expressed as mean ± SD (n = 6). ** $P < 0.01$.

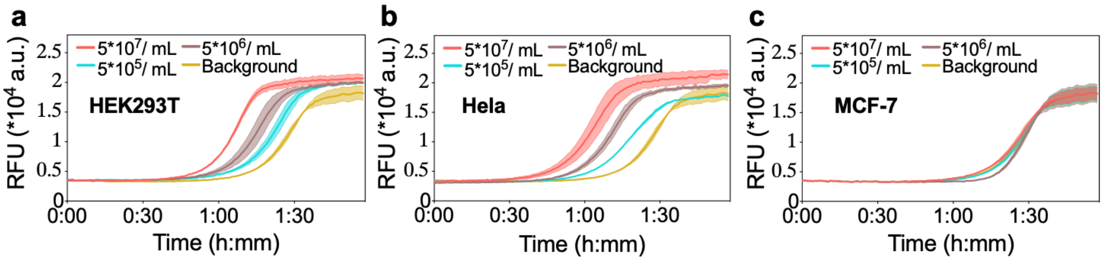

Supplementary Figure S29. Time-dependent fluorescence signals of CALSA in response to different tumor cell lines, including positive HEK293T (a) and Hela (b), as well as negative MCF-7 (c), at varying cell densities. Background represented samples without cell.

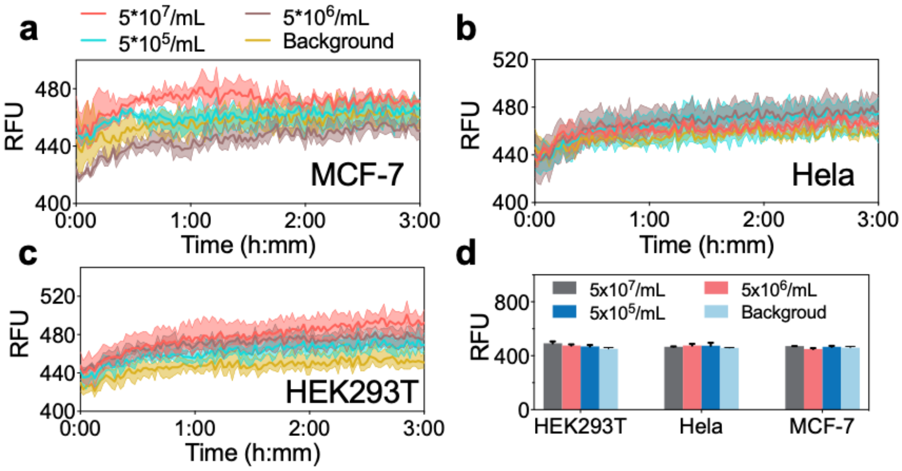

Supplementary Figure S30. The detection of cfDNA from different cell culture by the direct Cas12a detection method. Background represented samples without cell.
